# Selective Expansion of Viral Variants following Experimental Transmission of a Reconstituted Feline Immunodeficiency Virus Quasispecies

**DOI:** 10.1371/journal.pone.0054871

**Published:** 2013-01-23

**Authors:** Brian J. Willett, Martin Kraase, Nicola Logan, Elizabeth McMonagle, Mariana Varela, Margaret J. Hosie

**Affiliations:** 1 MRC-University of Glasgow Centre for Virus Research, University of Glasgow, Glasgow, United Kingdom; 2 Institut für Virologie, Veterinärmedizinische Universität Wien, Wien, Austria; University of Illinois at Urbana-Champaign, United States of America

## Abstract

Following long-term infection with virus derived from the pathogenic GL8 molecular clone of feline immunodeficiency virus (FIV), a range of viral variants emerged with distinct modes of interaction with the viral receptors CD134 and CXCR4, and sensitivities to neutralizing antibodies. In order to assess whether this viral diversity would be maintained following subsequent transmission, a synthetic quasispecies was reconstituted comprising molecular clones bearing *envs* from six viral variants and its replicative capacity compared *in vivo* with a clonal preparation of the parent virus. Infection with either clonal (Group 1) or diverse (Group 2) challenge viruses, resulted in a reduction in CD4+ lymphocytes and an increase in CD8+ lymphocytes. Proviral loads were similar in both study groups, peaking by 10 weeks post-infection, a higher plateau (set-point) being achieved and maintained in study Group 1. Marked differences in the ability of individual viral variants to replicate were noted in Group 2; those most similar to GL8 achieved higher viral loads while variants such as the chimaeras bearing the B14 and B28 Envs grew less well. The defective replication of these variants was not due to suppression by the humoral immune response as virus neutralising antibodies were not elicited within the study period. Similarly, although potent cellular immune responses were detected against determinants in Env, no qualitative differences were revealed between animals infected with either the clonal or the diverse inocula. However, *in vitro* studies indicated that the reduced replicative capacity of variants B14 and B28 *in vivo* was associated with altered interactions between the viruses and the viral receptor and co-receptor. The data suggest that viral variants with GL8-like characteristics have an early, replicative advantage and should provide the focus for future vaccine development.

## Introduction

Feline immunodeficiency virus (FIV) targets CD4+ helper T cells by an initial high affinity interaction between the viral envelope glycoprotein and CD134 (OX40) [1], [2] and a subsequent interaction with the chemokine receptor CXCR4 [3], [4]. As expression of CD134 is restricted to activated CD4+ (not CD8+) T cells, FIV infection of the domestic cat results in an immune dysfunction characterised by a progressive depletion of helper T cells. The resulting AIDS-like immunodeficiency manifests with chronic gingivitis and stomatitis, anorexia, cachexia, neurological signs and an increased incidence of malignancy.

There are distinct differences in pathogenicity amongst FIV strains. Infection with a cell culture-adapted strain of virus results in an inapparent infection with low viral loads and stable CD4+ T cell numbers [5]. In contrast, infection with a primary isolate of virus, serially passaged *in vivo* during the acute phase of infection, results in the development of a disease state characterised by a high viral load, precipitous decline in CD4+ T cell numbers, lymphoid depletion and susceptibility to opportunistic infections [6]. The pathogenicity of different strains of FIV may be influenced by both host and viral factors, for example variants bearing mutations in the FIV *orfA* gene are defective for growth in primary T cells [7]–[9] while the viral Vif protein permits evasion of the antiviral activities of host APOBEC proteins [10].

The surface glycoprotein Env is a primary determinant of cell tropism; in early infection the virus targets CD4+ helper T cells and macrophages primarily, while in later infection tropism extends to CD8+ T cells and B cells [11], [12] and it would appear that early and late isolates of virus may differ in the way they interact with the primary receptor CD134 and their propensity for CD134-independent infection [13], [14]. The virus-receptor interaction may evolve under the selective pressure of the humoral immune response; for example, hypervariation in the V5 loop of Env associated with escape from neutralising antibody alters the Env-CD134 interaction, increasing sensitivity to antagonism by anti-CD134 antibody while reducing sensitivity to inhibition by soluble CD134 [15]. Accordingly, selective pressure from the humoral immune response may alter the cell tropism and pathogenicity of the viral variants that evolve. Such “late” viral variants that emerge in FIV infected cats under the selective pressure of the humoral immune response may mirror the emergence of CXCR4-dependent (X4) variants of HIV with disease progression [16]. X4 variants emerge in approximately 50% of HIV-infected individuals, however, progression to AIDS may also occur in the absence of X4 variants suggesting that there is not a causal link between the presence of X4 variants and the development of AIDS. While the emergence of viral variants with the phenotype of “late” variants of FIV has been observed *in vivo* [15], it is not known whether these variants are less pathogenic, or whether they are transmitted poorly to naïve animals, analogous to the selective filtering of X4 variants of HIV following transmission (reviewed in [17]).

Identifying the strains of virus that are transmitted between cats is critical to the selection of strains for future formulations of FIV vaccines. While a commercial FIV vaccine has been developed and has demonstrated a degree of efficacy [18]–[22], the vaccine failed to protect cats from challenge with the prototypic United Kingdom subtype A strain, GL8 [23]–[27]. If GL8 is representative of field strains of FIV, then the incorporation of antigens from GL8-like viruses into the next generation of FIV vaccines would seem prudent.

In previous studies, we followed experimental infection of cats with a molecular clone of GL8 for a period of six years [5], [28]. At the end of the study, we observed the establishment of pools, or “swarms” of viral variants, each with distinct receptor usages and sensitivities to neutralising antibodies [15], [29]. Included within these viral quasispecies, were viruses that were near identical to the parent GL8 strain of virus [29]. As FIV is thought to be transmitted between cats by fighting and biting, the entire pool of viral variants could, potentially, be transmitted between cats, circumventing barriers to transmission such as mucosal surfaces [17]. In this study we set out to investigate what would happen to a diverse GL8-derived inoculum following experimental transmission. Would diversity be maintained or would selected viral variants dominate? Here we demonstrate that following transmission of a pool of viral variants, GL8-like viruses dominate and appear to have a replicative advantage.

## Materials and Methods

### Ethics statement

This study was approved by the University of Glasgow Institutional Animal Care and Use Committee (OLAW Assurance A5181-01) “The control of feline retroviral infection in domestic cats” and was conducted under licence from the UK Government Home Office under the Animals (Scientific Procedures) Act 1986. The use of recombinant viruses was approved by the Health and Safety Executive under The Genetically Modified Organisms (Contained Use) Regulations 2000.

### Cells and viruses

MYA-1 [30], MCC-CD134 [1] and CLL-CD134 [13] cells were cultured in RPMI 1640 medium, while HEK-293T were maintained in Dulbecco's modification of Eagle's medium (DMEM). Media for HEK-293T, MCC-CD134 and CLL-CD134 were supplemented with 400 µg/ml G418. All media were supplemented with 10% foetal bovine serum (FBS), 2 mM glutamine, 0.11 mg/ml sodium pyruvate 100 IU/ml penicillin, 100 μg/ml streptomycin (complete medium). The medium for MYA-1 cells was supplemented with conditioned medium from a murine cell line (L2.3) transfected with a human IL-2 expression construct (M. Hattori, University of Tokyo, Japan) at a final concentration equivalent to 100 U/ml of recombinant human IL-2, and 50 µM 2-mercaptoethanol. All media and supplements were obtained from Invitrogen Life Technologies, Paisley, UK.

### Experimental design

Six years post-infection of cat A613 with a molecular clone of the GL8 strain of FIV, a viral quasispecies had evolved comprising diverse variants with distinct biological properties, including resistance to virus neutralising antibody and discernible differences in the nature of the virus-receptor interaction [5], [29]. In this study, we compared transmission of a reconstituted quasispecies with transmission of the cloned virus. In order to prepare a defined “quasispecies”, *env* genes from five distinct variants (B14, B19, B28, B30, and B31 [15]) and a single variant identical to the parent GL8 clone (B32) were amplified and sub-cloned into the GL8(MYA) molecular clone. Our aim was to reconstitute a quasispecies representative of that isolated from cat 613 at post-mortem and comprising variants with distinct sensitivities to either neutralising antibody, soluble CD134 (sCD134) or anti-CD134 antibody (7D6), and which had shown variations in the way they utilised CD134 as a receptor [13], [14] (summarised in Fig. S1 and shown in detail in Figure S2).

Challenge viruses were prepared by transfecting the six variants into HEK-293T cells and recovering into primary IL2-dependent CD4+ T cells (MYA-1 cells). MYA-1 cells express CD4, CXCR4 and CD134 at similar levels to mitogen-stimulated helper T cells and support the replication of all strains of FIV tested to date without applying a selective pressure to viruses cultured therein. Stocks of each virus were 0.45 µm-filtered and frozen at −80°C. Viral titres were quantified by serial dilution upon uninfected MYA-1 cells and calculated using the Kärber formula, monitoring the cells visually for cytopathicity and for the production of FIV p24 by enzyme-linked immunosorbent assay (ELISA, PetCheck FIV antigen ELISA, IDEXX Corp., Portland, Maine, USA). Reverse transcriptase activity within each viral stock was estimated by non-isotopic RT assay (Cavidi AB, Uppsala, Sweden).

Previous studies have demonstrated that four animals per group would be sufficient to distinguish differences in the biological phenotypes of the challenge viruses [5]. Accordingly, two groups of four age-matched specific pathogen free animals were infected with 10,000 TCID50 of either the parent molecular clone of GL8-B32 (animals 821, 822, 823 and 824) or a pool containing equal TCID50 of six variants bearing the *env* genes of B14, B19, B28, B30, B31 and B32 to a final combined challenge dose of 10,000 TCID50 (animals 825, 826, 827 and 828). Thus, Group 1 received 10,000 TCID50 of clonal virus, while Group 2 received 10,000 TCID50 of the quasispecies (Figure S3). Animals were infected intra-peritoneally and blood samples collected at three-weekly intervals. EDTA-anti-coagulated blood samples were processed for flow cytometry while plasma was stored for immunoblotting. White blood cells were prepared using whole blood lysis and frozen immediately at −80°C for subsequent analysis of proviral loads. At post-mortem, 21 weeks post-infection, lymphoid tissues were collected and processed, the separated cells were then cryo-preserved for subsequent ELISpot analyses.

### Flow cytometry

Anti-feline CD4-FITC (vpg34), anti-feline CD8αβ-PE (vpg9) and anti-human CD14-FITC (TÜK4) were obtained from AbD Serotec Ltd., Oxford, U.K. EDTA anti-coagulated blood was processed for flow cytometric analyses by whole blood lysis as described previously [31]. Samples were analysed on a Beckman Coulter EPICS MCS-XL flow cytometer, 10,000 events being collected for each sample in LIST mode. Data were processed using EXPO 32 ADC Analysis software (Advanced Cytometry Systems).

### Immunoblotting

Pooled supernatants from FIV GL8-infected MYA-1 cells were filtered at 0.45 µm and virus pelleted by ultracentrifugation at 28,000 rpm in an SW28 rotor on a Beckman L8-70 ultracentrifuge. The pelleted virus was resuspended in reducing Laemmli sample buffer [32] and separated on 4–15% polyacrylamide gels. Separated proteins were transferred to nitrocellulose by electroblotting (iBlot™, InVitrogen Life Technologies, Paisley, UK) and viral antigens were detected using either cat plasma samples or control pooled polyclonal cat plasmas from either uninfected or FIV GL8-infected cats. Bound antibodies were detected using biotinylated goat anti-cat IgG (Vector Laboratories Ltd., Peterborough, UK), while bound conjugate was revealed using the Vectastain ABC kit and 5-bromo-4-chloro-3-indolyl phosphate/nitroblue tetrazolium substrate (Vector Laboratories Ltd.).

### Virus neutralising antibody (VNA) assays

FIV *env* gene expression constructs have been described previously [1], [13], [14]. 5 µg of each VR1012-*env* construct and 7.5 µg of pNL4-3-Luc-E^−^R^−^ were co-transfected into HEK-293T cells using SuperFect activated dendrimer (QIAgen, Crawley, UK) as per manufacturer's instructions. The nomenclature “HIV(FIV)” denotes an FIV Env protein on an HIV particle. Culture supernatants were collected at 72 hours post-transfection, filtered at 0.45 µm and frozen at –80°C until required. Target cell lines were seeded at 5×10^4^ cells per well of a CulturPlate™-96 assay plate (Perkin Elmer, Life and Analytical Sciences, Beaconsfield, UK) and used immediately. The cells were then infected with 50 µl of HIV (FIV) luciferase pseudotypes, cultured for 72 hours and then luciferase activity quantified by the addition of 100 µl of Steadylite HTS™ (Perkin Elmer) luciferase substrate prior to measurement by single photon counting on a MicroBeta TriLux luminometer (Perkin Elmer).

Plasmas were diluted 5-fold in MYA-1 culture medium and then 25 µl of each dilution (in triplicate) was incubated with 25 µl of HIV(FIV) luciferase pseudotype (containing ∼50 pg of reverse transcriptase (RT) activity), incubated for one hour at 37°C and then added to 50 µl (5×10^4^ cells) of MYA-1 cells per well of a CulturPlate^TM^-96 assay plate (Perkin Elmer). The cells were then cultured for 72 hours and luciferase activity quantified by the addition of 100 µl of Steadylite HTS^TM^ (Perkin Elmer) luciferase substrate and measurement on a MicroBeta TriLux luminometer. Percent neutralisation was calculated by comparing the mean luciferase counts at each plasma dilution with the mean luciferase counts for the no plasma control.

### Inhibition of viral entry

1×10^5^ MYA-1, CLL-CD134 or MCC-CD134 cells were incubated with AMD3100 in complete medium in triplicate wells of 96-well culture-treated luciferase assay plates (CulturPlateTM 96) for 30 minutes at 37°C. HIV(FIV) pseudotypes were then added and the plate returned to the 37°C incubator. Cultures were maintained for 72 hours post-infection at which point 100 µl of Steadylite HTSTM (Perkin Elmer) luciferase substrate was added and luminescence measured by single photon counting on a MicroBeta luminometer (Perkin Elmer). Percent infection was calculated by comparing the mean (n = 3) luciferase activity of each antagonist concentration against the mean (n = 3) luciferase activity of untreated cells (100% infection).

### Quantification of proviral loads by real time PCR

Primers and probes were purchased from Eurofins-MWG. DNA was prepared from peripheral blood mononuclear cells (PBMC) and stored in Tris-EDTA buffer prior to analysis. For total viral loads, a *gag* PCR was utilized [33] whereby 400 ng of each sample was added to Taqman mastermix (Applied Biosystems) containing 20 pmol of forward and reverse primers FIV1360F (5′-GCA GAA GCA AGA TTT GCA CCA-3′) and FIV1437R (5′-TAT GGC GGC CAA TTT TCC T-3′) and 10pmol probe FIV1416P FAM-TGC CTC AAG ATA CCA TGC TCT ACA CTG CA-TAMRA. Single viral variant analysis used a common reverse primer V5R1 (5′- GCT ACG GGG TTA TAC CAA TTT C-3′) and probe V5probe1 (5′- FAM-ATA GTG TTA AAA TGG CAT GTC CTA AAA ATC AAG GCA TCT-TAMRA-3′), in conjunction with a unique forward primer B14F (5′-GTA CAA ATA GTA GTA GTA CAA ACA GTA GT-3′), B19F (5′-ATA TGA ATT GTA ATT GTA CAA ATA GCA GTA CA-3′), B28F (5′-CAA ATA GTA GTA GTA CAA ATC GGC AAA-3′) or B30F (5′-GTA CAA ATA GTA GTA GTA CAA ATA GTA CA-3′). It was not possible to generate a unique V5 forward primer/probe combination for either clone B31 or the parent clone GL8 (B32) that could distinguish B31 or GL8 from the quasispecies as the V5 sequences of the viral variants were derived from GL8. A primer and probe set targeting V5 but which detected all variants with equal efficiency consisted of the primers G8V57829F (5′-GCA TTT CAA TAT GAC AAA AGC T-3′) and V5R1 and probe 5′-FAM-ATA GTG TTA AAA TGG CAT GTC CTA AAA ATC AAG GCA TCT-TAMRA-3′. Control amplifications used primers and probes adapted from ref. [33] and targeting 18 s rDNA; rDNA 343F (5′-CCA TTC GAA CGT CTG CCC TA-3′), rDNA 409R (5−TCA CCC GTG GTC ACC ATG-3′) and probe rDNA 370P (5′-FAM-CGA TGG TAG TCG CCG TGC CTA-TAMRA-3′). All estimations of viral load were adjusted to control for efficiency of 18 s rDNA amplification.

Prior to the analyses, the specificities and sensitivities of the variant-specific real-time PCRs were confirmed by comparing the ability of each primer and probe set to detect i) the homologous molecular clone in a background of cellular DNA and ii) the homologous molecular clone in a background of cellular DNA that had been spiked with an excess of the remaining five molecular clones. By comparing the performance of the assays under these conditions we were able to conclude that the B28 primer and probe set could detect 62 copies of B28 proviral DNA in the presence of 100,000-fold excess of the other five proviruses, at which time cross-talk between templates could be detected. The B30 primer and probe could also detect 62 copies of B30 but only remained specific in the presence of a 1000-fold excess of the other variants. The B14 primer and probe detected 62 copies with a reduced specificity of a 100-fold while the B19 primer and probe set proved the weakest of the four, with a sensitivity of 618 copies and a specificity of only 20-fold. While by no means optimal, the four primer and probe sets displayed sufficient specificity and sensitivity to facilitate a sound estimation of the composition of viral variants in peripheral blood. However, in order to confirm that the real-time PCR analyses were robust, we used limiting dilution PCR to amplify the V5 region of the *env* gene from PBMC DNA prepared from post-mortem blood samples from cats 825, 826, 827 and 828, the products were cloned and the nucleic acid sequences of approximately 50 clones from each animal were determined by capillary sequencing on an ABI3730xl DNA analyzer (Applied Biosystems) using BigDye® Terminator v1.1 cycle sequencing chemistry (Applied Biosystems).

### Quantification of cellular immunity by ELISpot

Lymph nodes were removed at post mortem and placed in ice-cold culture medium. Tissues were disrupted using crossed scalpels and cells separated by pipetting, washed by centrifugation and then purified by centrifugation through Ficoll-Paque™ (GE Healthcare UL Ltd, Little Chalfont, UK) separation medium. Lymphocytes were harvested from the interface, washed by centrifugation and stored in the vapour phase of liquid nitrogen in cryoprotectant consisting of culture medium supplemented with 20% FBS and 10% DMSO. Prior to ELISpot analysis, cryovials of cells were thawed rapidly at 37°C and washed in prewarmed culture medium. IFN-γ producing cells were enumerated using commercial IFN-γ ELISpot kits (R&D Systems, Abingdon, Oxford, UK) as per the manufacturer's protocol. 5×10^5^ cells were added to each well and incubated with 0.1, 1.0 or 10 µg (total protein concentration) of sucrose gradient-purified FIV GL8 that had been treated overnight with Aldrithiol^TM^-2 (AT-2, Sigma, Poole, UK). Control wells were incubated with medium alone or medium supplemented with 5 µg/ml Concanavalin A (Con A). Spot-forming cells were enumerated on an AID ViruSpot Reader, CADAMA Medical Ltd., UK. Subsequent analyses used pools of overlapping peptides derived from the predicted sequence of GL8 Env (15-mers overlapping by 10 amino acids and spanning the entire leader (L), surface (SU) and transmembrane (TM) domains) and which had been dissolved initially in HPLC-grade DMSO (Sigma-Aldrich, Poole, UK) and diluted in phosphate buffered saline pH 7.4 prior to use. All peptides were from Alta Biosciences (Birmingham, UK).

## Results

### Comparison of viral pathogenicity *in vivo*

**Figure 1:**
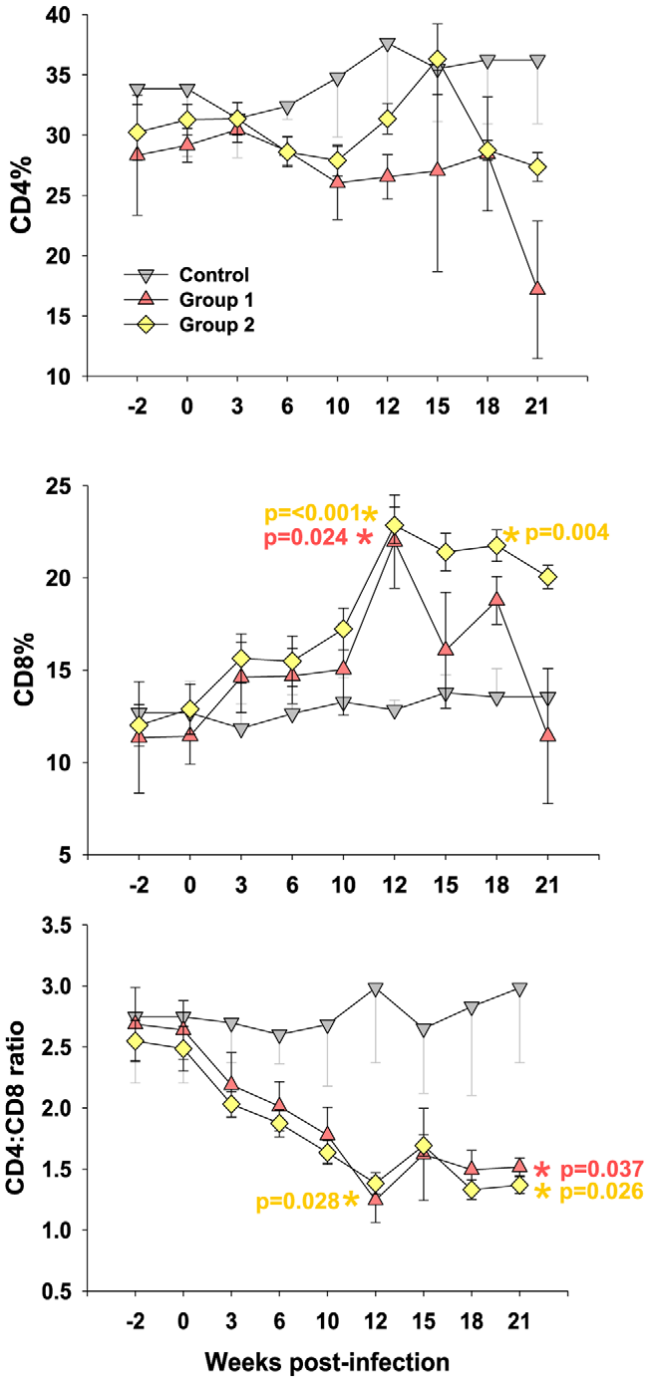
Immunological alterations following FIV infection. Cats in Group 1 were challenged with clonal GL8 (variant B32), cats in group 2 were challenged with the reconstituted quasispecies challenge (B14, B19, B28, B30, B31 and B32) and control cats remained unchallenged. Percentages of lymphocytes expressing (A) CD4 and (B) CD8 were measured by flow cytometry and are shown as group means +/− SE. (C) CD4:CD8 ratios were calculated from the percentage of CD4 and CD8 positive lymphocytes and are displayed as group means +/− SE. p-values for statistically significant differences between infected and control groups are shown (Student's *t*-test).

The replication of cloned GL8 (Group 1) *in vivo* was compared with the reconstituted quasispecies (Group 2). While, infection of both groups led to a significant reduction in the CD4:CD8 ratio compared with age-matched controls (Fig. 1), there was no significant difference in CD4:CD8 ratios between the two groups at each of the time points that were compared. The reduced CD4:CD8 ratios derived primarily from a reduction in CD4+ T lymphocytes coupled with a significant increase in CD8+ T lymphocytes as described previously [31]. This expansion of CD8+ lymphocytes reached a maximum by 12 weeks post-infection and was sustained in group 2 animals but was transient in group 1 animals, falling to the control level by 21 weeks post-infection. By 21 weeks post-infection, CD4+ lymphocytes were depleted in both study groups, but most markedly in group 1. Thus while infection with the clonal virus appeared to induce a transient increase in CD8+ lymphocytes and significant reduction in CD4+ lymphocytes, infection with the mixture of viral variants induced a sustained increase in CD8+ lymphocytes and a more modest reduction in CD4+ lymphocytes.

**Figure 2:**
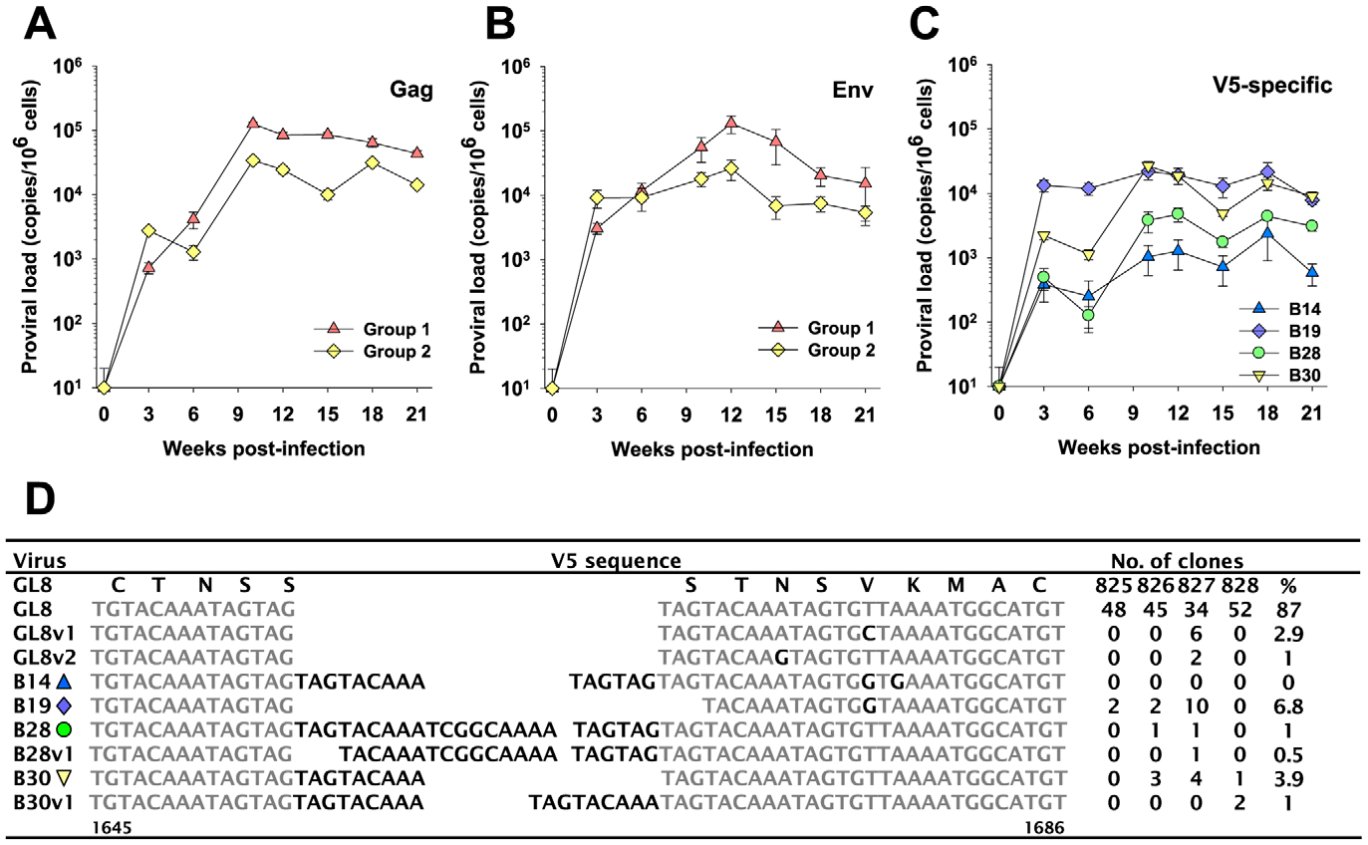
Proviral loads following FIV infection. Mean proviral loads (+/−SE) for groups 1 (clonal challenge) and 2 (quasispecies challenge) were estimated by real-time PCR using primers derived from either (A) *gag* (all clones shared a common *gagpol* sequence) or (B) a conserved sequence within the V5 region of *env*. (C) The mean proviral loads (+/−SE) for individual variants B14, B19, B28 and B30 in study group 2 were estimated using real-time PCR targeting unique determinants within the V5 region of *env*. All estimates of proviral load were performed in triplicate. (D) V5 *env* sequences amplified at 21 weeks post-infection from cats 825, 826, 827 and 828 infected with the reconstituted quasispecies. The region spanning nucleotides 1640 to 1694 of the GL8 *env* open reading frame is shown, the predicted amino acid translation of this region from the parent GL8 strain is shown above the parent GL8 sequence. The number of clones isolated with the corresponding V5 sequence is listed; no clones bore the B14 sequence, consistent with the poor replication of B14 *in vivo*.

Next, we compared the proviral loads in peripheral blood mononuclear cells (PBMC) between the two groups. As both groups of cats were infected with viruses derived from the GL8 molecular clone, primers and probes for real time PCR were designed that targeted the common *gag* sequence shared by all viruses. When the 21-week area under the curve was compared no statistically significant differences were found between groups 1 and 2, both groups achieving peak proviral loads approaching 10^5^ proviral copies/10^6^ cells. However, in general, the group 1 cats infected with the homogeneous GL8 preparation achieved higher absolute viral loads than group 2 infected with the quasispecies (Fig. 2A), detectable by three weeks post-infection and peaking by 10 weeks post-infection. The sole exception was three weeks post-infection where the viral loads in group 2 were higher than those in group 1. Overall, even though the inocula were adjusted to ensure that the animals were challenged with matched TCID50s, the Group 2 inoculum with increased *env* diversity appeared to replicate more poorly *in vivo*.

To confirm the validity of these findings, DNA was extracted from a fresh set of PBMC samples and the analyses repeated using primers and probes derived from a conserved stretch of the *env*. This completely independent analysis (Fig. 2B) recapitulated the findings with the *gag* primers and probe, demonstrating higher proviral loads in the group 1 cats with the single exception of week 3, at which time point the situation was reversed. In conjunction with the more pronounced reduction in CD4+ T lymphocytes, the higher viral loads detected in the group 1 animals is consistent with a higher viral load being linked to a more profound depletion of CD4+ lymphocytes.

Next, we asked whether the lower viral load in the group 2 animals resulted from a defective ability to replicate *per se*, or whether individual variants within the group 2 mixture were affected more markedly than others. Despite the high degree of sequence similarity amongst the six molecular clones comprising the quasispecies, there was sufficient variation in the V5 region to permit an estimation of the proviral loads of four of the individual clones by real-time PCR using selective probe and primer sets (Fig. 2C). These primers and probes did not detect the GL8-like clones B31 and B32 (as the variant V5s were derived largely from sequence repetition, it was not possible to detect B31 and B32 specifically in the presence of a background of B14, B19, B28 and B30). Statistically significant differences were found in the 21-week area under the curve among the four clones we were able to discriminate (ANOVA p<0.05). B19 achieved a viral load of ∼10^4^ copies/10^6^ cells by three weeks post infection and this level was maintained throughout the course of the study. B30 also achieved a load of 10^4^ copies/10^6^ cells although not until 10 weeks post-infection, suggesting replication may have been slightly retarded. The most significant differences were the lower loads achieved by B28 and, more strikingly, B14; both clones achieved substantially lower viral loads (∼10^3^ copies/10^6^ cells for B14) in spite of matched TCID50 of all six viruses having been included in the inoculum. Real time analyses of the number of viral genomes within each of the individual virus stocks used to prepare the challenge inocula revealed that each of the individual viral stocks contributed ∼10^9^ viral genomes (range 1.3 to 6.0×10^9^) to the final challenge stock following adjustment for *in vitro* titre. Thus the marked disparity in viral loads observed *in vivo* could not be accounted for by disparities in the number of viral genomes in the challenge stocks. Specifically, the B14 stock contributed 2.2×10^9^ genomes to the challenge stock while B32 contributed 1.3×10^9^ genomes. The data suggest that the challenge stock for the group 2 cats had broadly similar numbers of genomes from each individual virus and that the lower loads for B14 detected *in vivo* reflected a failure of this viral variant to thrive *in vivo*.

Given the sensitivity and specificity of the real-time analyses (see Methods), we can conclude that estimates of B28 and B30 copy number are likely to be accurate; the total viral load in the group 2 cats (Fig. 2B) did not exceed ∼10^4^ copies and thus there would have been insufficient competing templates to interfere with the quantification of these variants. However, given that lentiviruses acquire mutations with successive cycles of replication and that such mutations may impact upon the accuracy of the real-time PCR analyses, we performed a second analysis in which the V5 region of *env* was amplified directly from blood samples collected at post mortem from the four animals infected with the quasispecies (825, 826, 827, 828). The V5 amplicons were then cloned and their nucleic acid sequences determined (Fig. 2D). The majority of the amplicons sequenced from each of the four animals had V5 sequences identical to that of variants B31 and B32 (87% of total, with two additional closely related variants, GL8v1 and GL8v2, identified in animal 827), suggesting that by 21 weeks post-infection the GL8-like variants were dominant in peripheral blood. Variants B19 and B30 were detected at a lower frequency (6.8% and 3.9%). Real-time PCR analysis had suggested that variants B14 and B28 were present at significantly lower levels than the other viral variants and this was borne out by the sequencing analysis in that we were unable to detect B14 by direct sequencing while B28-like variants were detected on only three occasions. The data are consistent with the selective replication of GL8-like variants during the early phase of infection.

### Role of neutralising antibodies in suppressing viral replication

**Figure 3:**
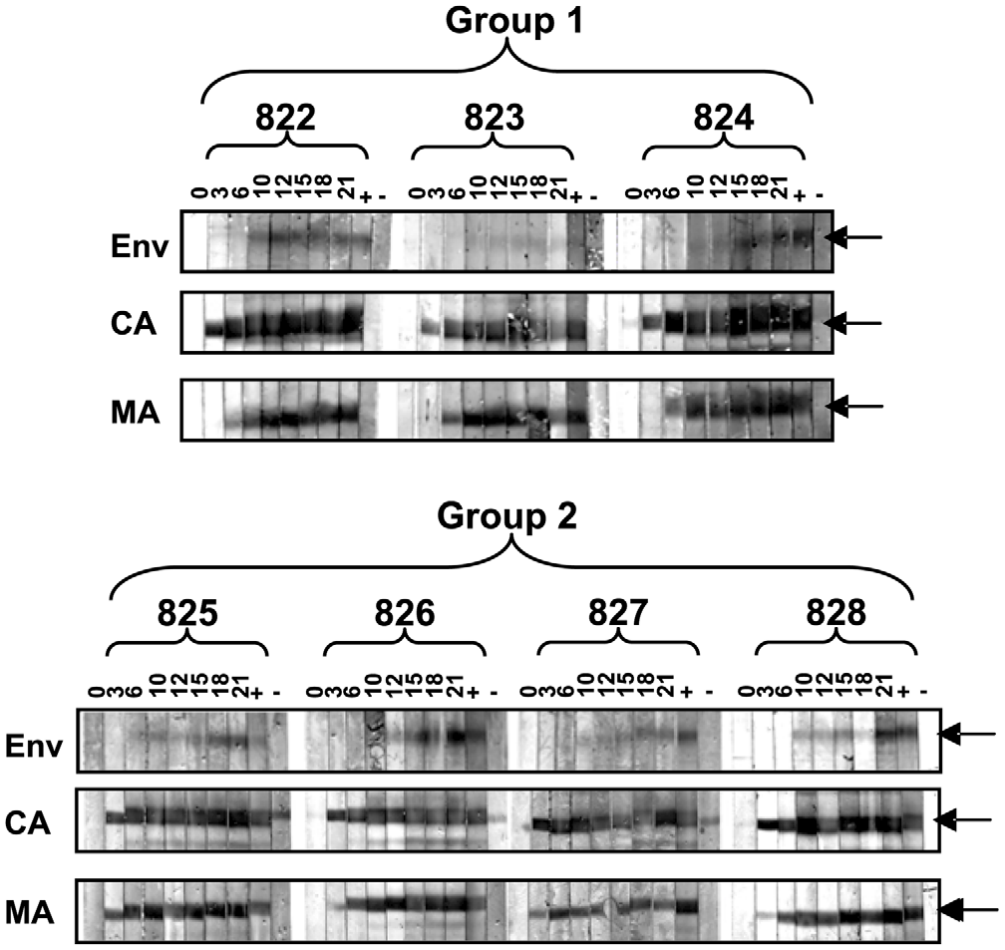
Serological response to FIV infection. Plasmas were collected from cats in group 1 (822, 823, 824) and group 2 (825, 826, 827 and 828) at 0, 3, 6, 10, 12, 15, 18 and 21 weeks post-infection and were screened by immunoblotting against purified GL8 virus. Also shown are positive (+) and negative (−) control plasma samples. Bands corresponding to matrix (MA) and capsid (CA) proteins, and the envelope glycoprotein (Env) are indicated by arrows.

Pressure from the humoral immune response in the form of virus neutralising antibody selects for the emergence of viral variants bearing mutations in V5 that facilitate escape from neutralisation and which may alter the nature the virus-receptor interaction [15]. It is possible therefore that the replication of the B14 and B28 variants was suppressed by the humoral immune response although given that replication of these variants was lower as early as 3 weeks post-infection this would seem unlikely. To determine whether antibody-mediated neutralisation played a role in the reduced replicative capacity of B14 and B28, we first established whether the cats had mounted an anti-Env antibody response within the 21 week study period. Sequential samples of plasma from the infected animals were used to probe immunoblots prepared from pelleted IL2-dependent T cell-grown FIV GL8 (Fig. 3). Sero-reactivity against the viral capsid protein p24 (CA) could be detected as early as three weeks post-infection followed by a response against the matrix protein p17 (MA) at 6 weeks post-infection. Consistent with previous findings, the humoral response to Env developed more slowly and in most cats was not detectable until 10 to 12 weeks post-infection. At the time of post-mortem, 21 weeks post-infection, all animals had mounted a serological response against viral proteins, including antibodies against Env.

**Figure 4:**
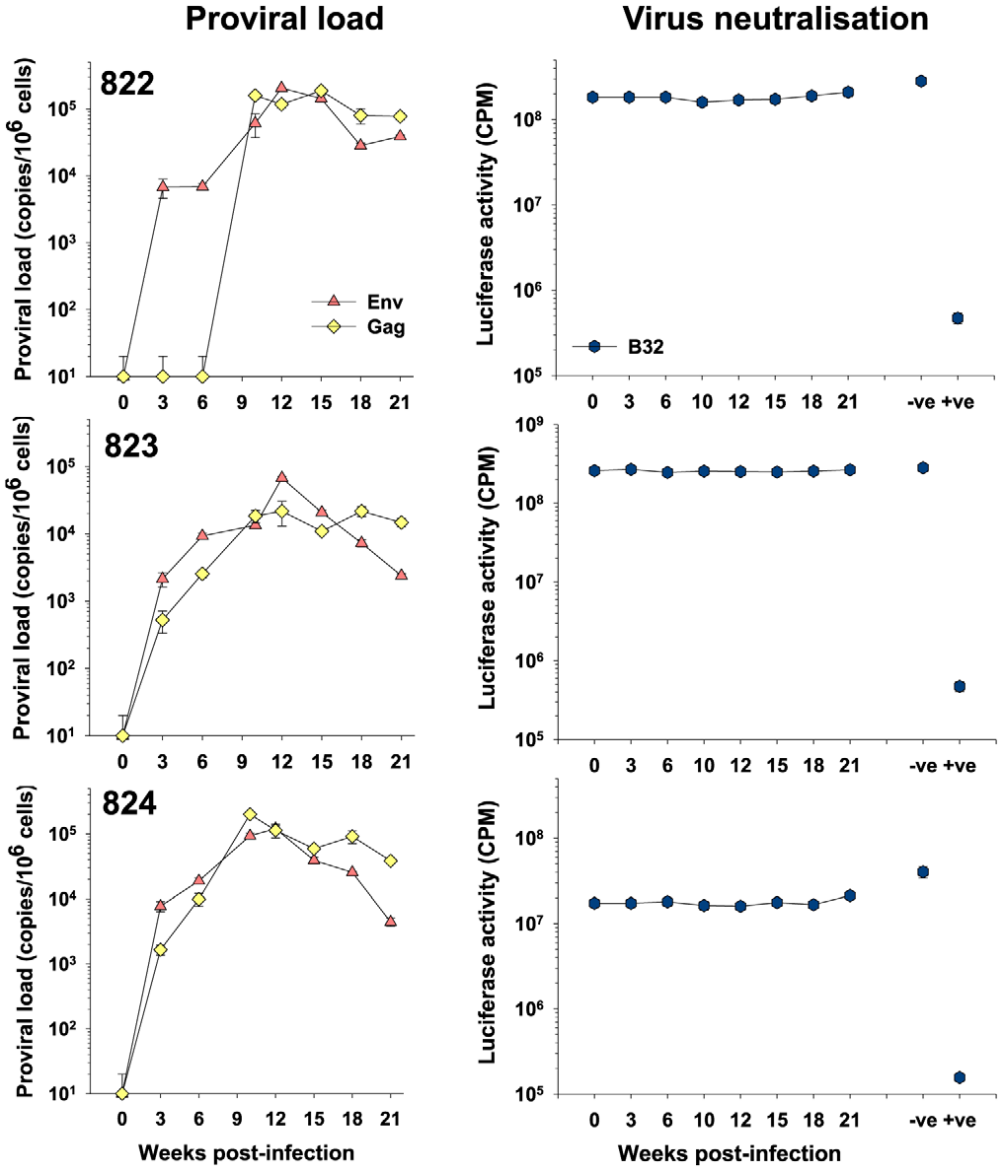
Absence of neutralising antibodies in early FIV infection – Group 1. Sequential plasmas from group 1 (822, 823 and 824) animals collected at 0,3,6,10,12,15,18 and 21 weeks post-infection were diluted 1∶100 and assayed for the ability to neutralise HIV(FIV) pseudotypes bearing the B32 Env. Also shown are pooled uninfected (−ve) plasma and post-mortem plasma from A613 (+ve). Neutralisation data are shown in comparison with total proviral loads for each cat as estimated using either *gag* or *env*–specific real-time PCR. All estimates of proviral load and luciferase activity are displayed as mean (n = 3) +/− SE.

Having confirmed the presence of Env-specific antibodies we next asked whether virus neutralising antibodies contributed to the humoral response and whether the strength of the neutralising antibody response correlated with changes in the viral loads of individual animals. Firstly, we examined neutralisation of the clonal virus (B32) by homologous antibody from the Group 1 animals. While the PM plasma from cat 613 (+ve control) neutralised homologous virus effectively, reducing infectivity by over 100-fold (Fig. 4), no virus neutralizing antibodies were detected in the sequential plasmas from animals 822, 823 and 824 (infected with the clonal inoculum), even though there was clear evidence of a downward trend in viral load in cats 823 and 824 between 12 and 21 weeks post-infection. While all neutralisation assays were performed with a level of input virus that displayed marked neutralisation by plasma from cat 613, we considered the possibility that very weak neutralising antibodies may be present in the sequential plasmas and that these were masked by the level of input virus. Accordingly, the neutralisation assays were repeated with 10-fold and 100-fold reductions in the level of input viral pseudotype, however, no neutralising activity was revealed in the plasma samples irrespective of the level of input virus (not shown), confirming that neutralising antibodies were not present in the sequential samples.

**Figure 5:**
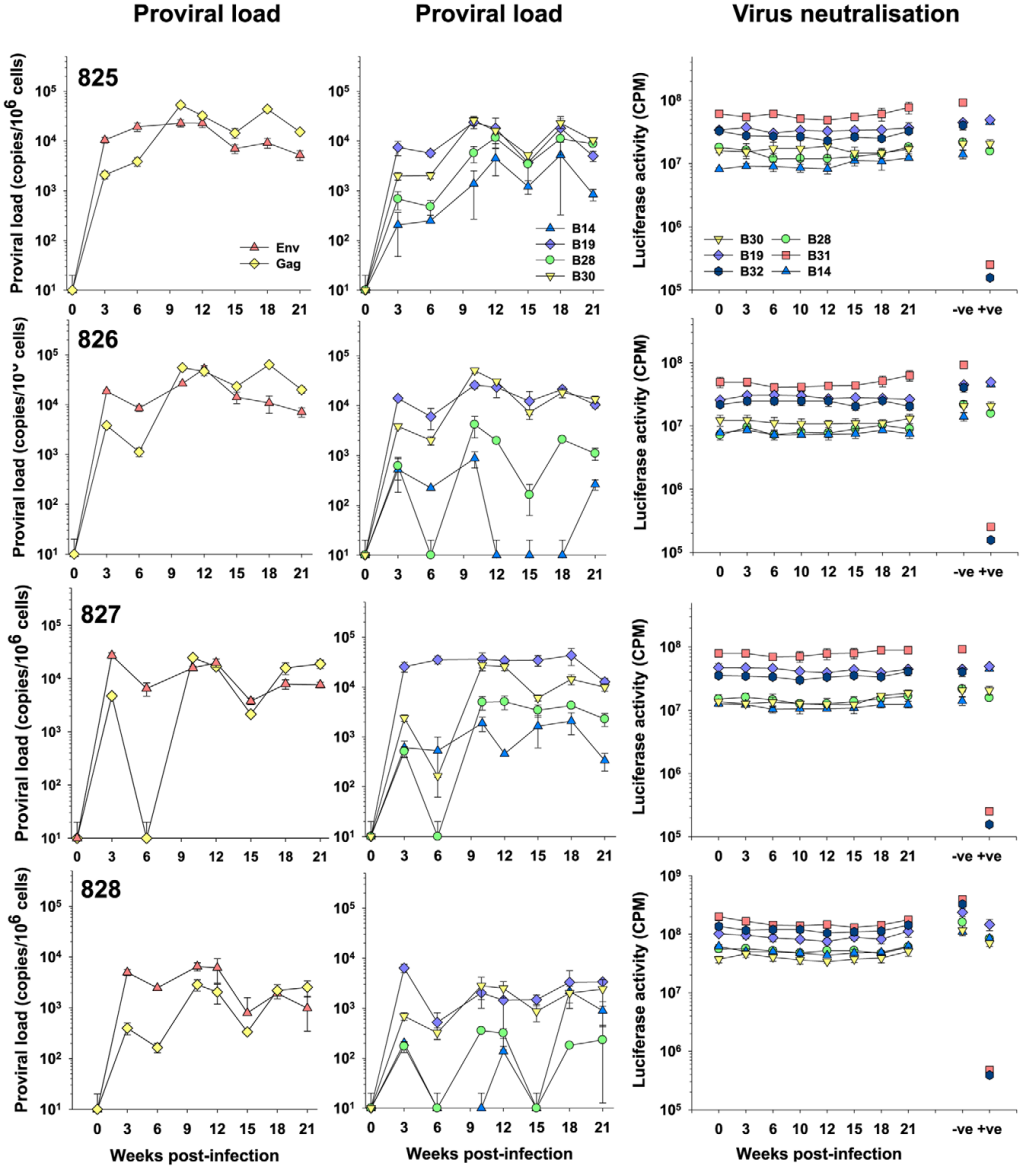
Absence of neutralising antibodies in early FIV infection – Group 2. Sequential plasmas from group 2 (825, 826, 827 and 828) animals collected at 0,3,6,10,12,15,18 and 21 weeks post-infection were diluted 1∶100 and assayed for the ability to neutralise HIV(FIV) pseudotypes bearing the B14, B19, B28, B30, B31 and B32 Envs. Also shown are pooled uninfected (−ve) plasma and post-mortem plasma from A613 (+ve). Neutralisation data are shown in comparison with total proviral loads for each cat as estimated using either *gag* or *env*–specific real-time PCR, or the variant (B14, B19, B28 and B30)-specific V5-targeted real-time PCR. All estimates of proviral load and luciferase activity are displayed as mean (n = 3) +/− SE.

We next investigated the neutralisation of the individual viral variants in the group 2 cats (Fig. 5). As each animal had been infected with a pool of six viral variants, sequential plasma samples from each of the four animals were screened for neutralising activity against HIV(FIV) pseudotypes bearing Envs from each of the viral variants. Again, no neutralising activity could be detected in any of the plasmas, while the positive control plasma from cat 613 neutralised the GL8-like variants B31 and B32 effectively. Variants B14 and B28, which grew poorly *in vivo*, resisted neutralisation by the sequential plasmas from all four animals. Again, reducing the level of input virus in the assay did not reveal the presence of weak neutralising antibody activity. As no correlation was noted between proviral load and neutralising antibody activity, the data do not support the involvement of neutralising antibody in restricting the early growth of variants such as B14 and B28.

### Cellular response to viral infection

**Figure 6:**
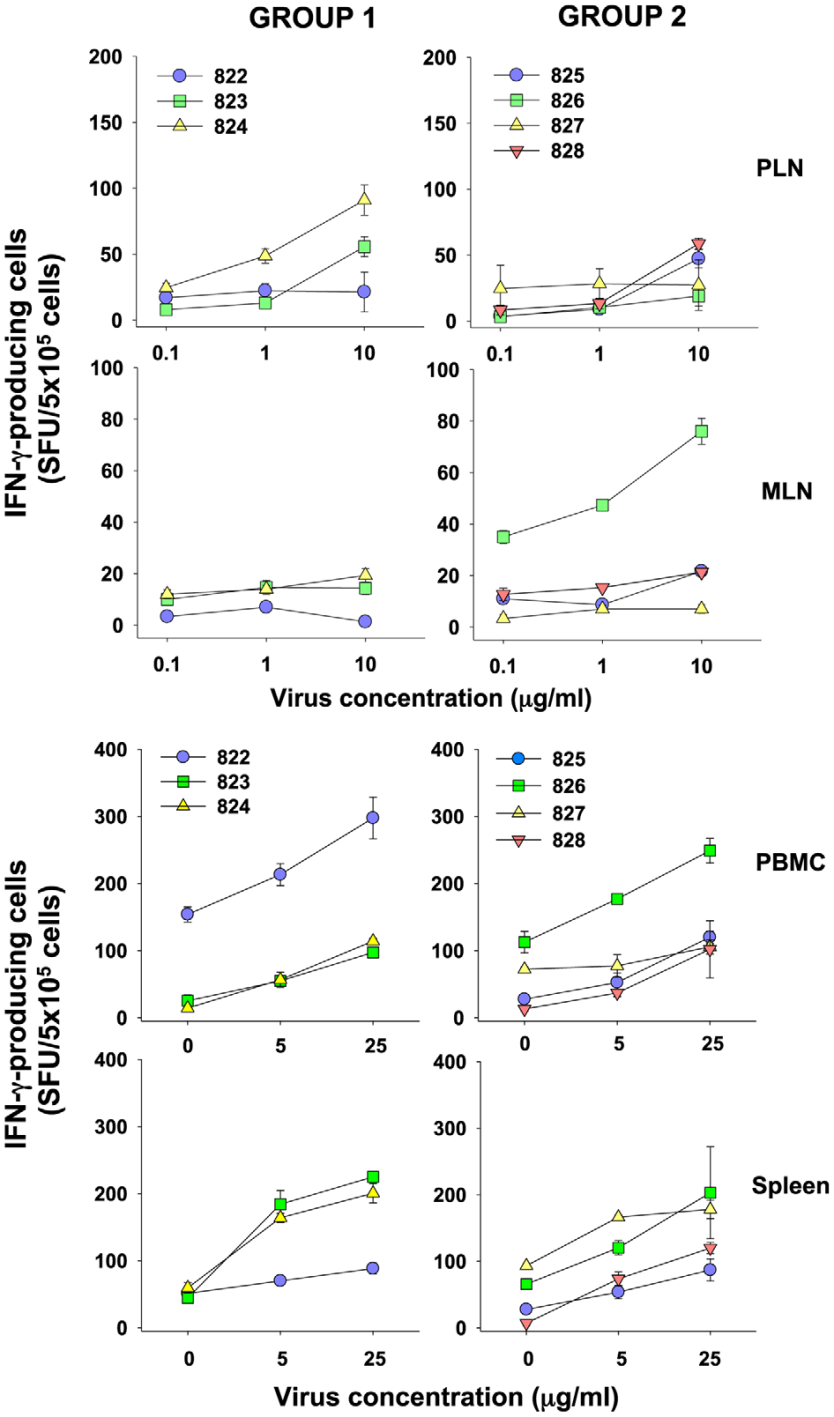
Cellular immune response in FIV infected cats. Cells from popliteal (PLN) or mesenteric (MLN) lymph nodes, peripheral blood (PBMC) or spleen were screened for interferon-**γ** production by ELISpot using AT2-inactivated virus as the source of viral antigen (each point represents the mean +/−SE of triplicate wells).

The cellular response to HIV infection develops during the acute phase of viral replication and is thought to contribute significantly to the reduction in viral load [34], [35]. Moreover, HIV-1 specific CD8+ T cells have been detected prior to the detection of a humoral response [34]–[37]. The acute phase of FIV infection is accompanied by the rapid expansion of a CD8+ lymphocyte population that is thought to represent an activated T cell population responding to virus replication [31], [38], [39]. Both Group 1 and Group 2 cats displayed such an expanded population (Fig. 1) indicating that a cellular immune response may have been induced in response to viral replication. Although our understanding of cellular immunity in the cat is limited, reagents have now been developed with which interferon-γ (IFNγ) ELISpot assays may be performed [40], [41]. We therefore examined lymphoid tissues (popliteal and mesenteric lymph nodes, and spleen) and peripheral blood mononuclear cells collected at post mortem for evidence of a cellular immune response using Aldrithiol™-2 (AT2)-inactivated whole GL8 virus as the source of viral antigen. Responses varied between cats and between tissues (Fig. 6). Amongst the cats infected with the clonal virus preparation (group 1), a strong response was detected in the PBMC of cat 822, while responses were not detected in either popliteal or mesenteric lymph node samples and a weak response was observed in spleen. In contrast, responses were detected in the spleen, blood and popliteal lymph nodes of cats 823 and 824.

IFN-γ-producing cells were detected in the spleens and PBMCs of animals 825, 826, 827 and 828 challenged with the quasispecies (group 2), but were less evident in the mesenteric and popliteal lymph nodes, with the notable exception of 826 in which marked virus-specific responses were evident in not only spleen, but also both PBMC and mesenteric lymph node. It is notable that 826 displayed very low viral loads for individual variants B14 and B28 perhaps suggesting an involvement of the cellular immune response in suppressing viral replication in this animal.

**Figure 7:**
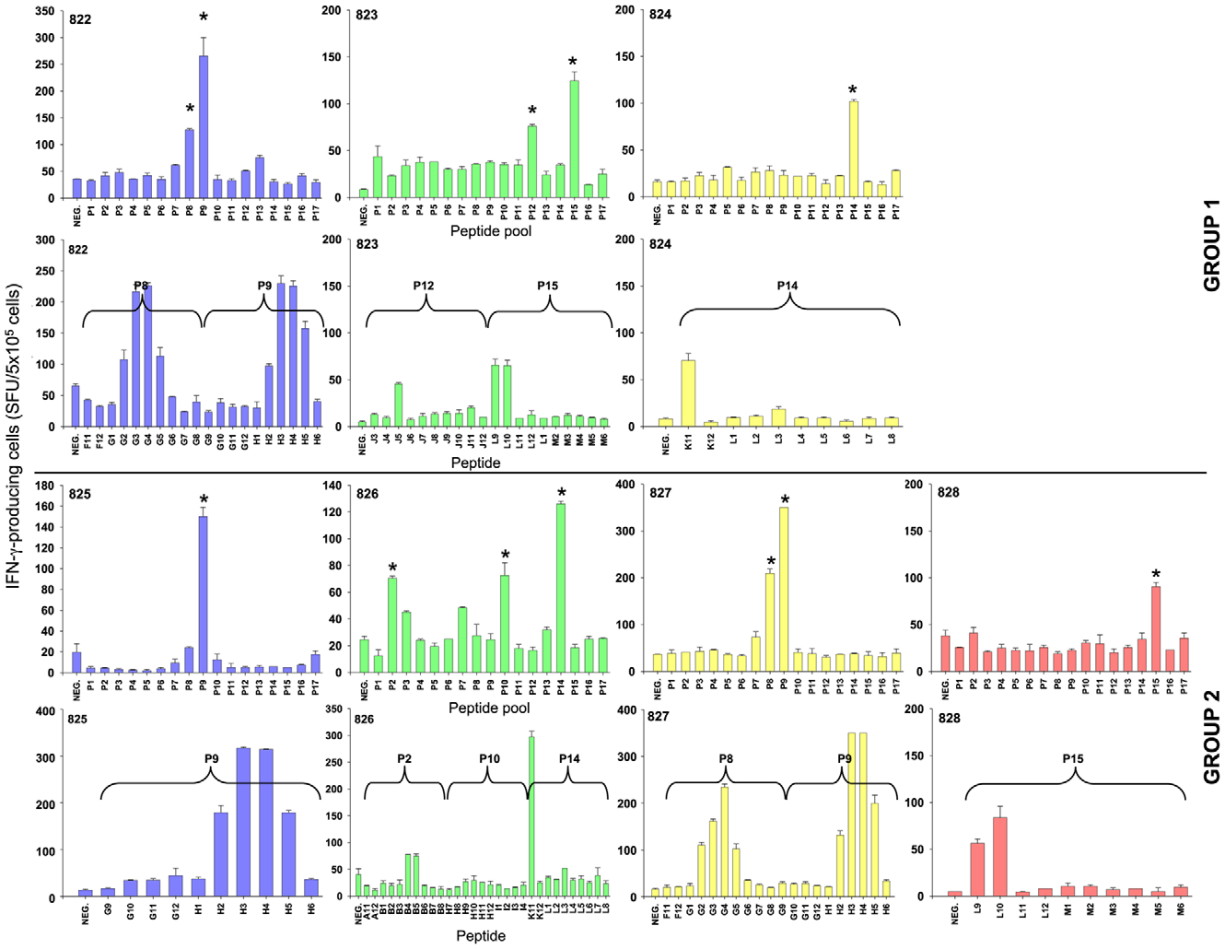
Epitope mapping of the cellular immune response in FIV infected. T cell epitopes were mapped using spleen cells from 822,823,824,825,826,827 and 828. Analyses used 17 pools of 10 x 15-mer peptides, each overlapping by 10 amino acids (pools P1-P17). Representative ELISpots for spleen cells from animal 822 against peptide pools P6 to p10) are included. All animals were screened with 17 peptide pools and where strong responses were confirmed (*), individual epitopes were then identified by splitting pools into their component peptides and repeating the analyses.

Given the robust cellular responses detected in spleen cells from the infected cats, we attempted to map the determinants in Env recognised by the responding cells. As all animals were infected with viruses that were identical in all genes except *env*, we reasoned that any specificity in the cellular response for individual viral variants would have to target Env. Accordingly, spleen cells from animals in groups 1 and 2 (Fig. 7) were screened against 17 pools of 10 overlapping 15-mer peptides representing the entire protein encoded by the *env* gene. Positive pools were identified and the analyses repeated using each of the individual peptides comprising the positive pool. Cellular responses to the pooled peptides were generally stronger than to inactivated virus as would be expected due to enrichment of individual peptides. Using this strategy we established that among the group 1 animals, pools P8 and P9 were recognised by 822, pools P12 and P15 by 823 while pool P14 alone elicited a response from 824 (Fig. 7). In contrast, among the group 2 animals, P9 stimulated a response from 825, three pools P2, P10 and P14 were recognised by 826, pools P8 and P9 were targeted by 827, while 828 responded to a single pool, P15 (Fig. 7). The data are consistent with the early cellular response targeting a limited number of epitopes in Env.

**Figure 8:**
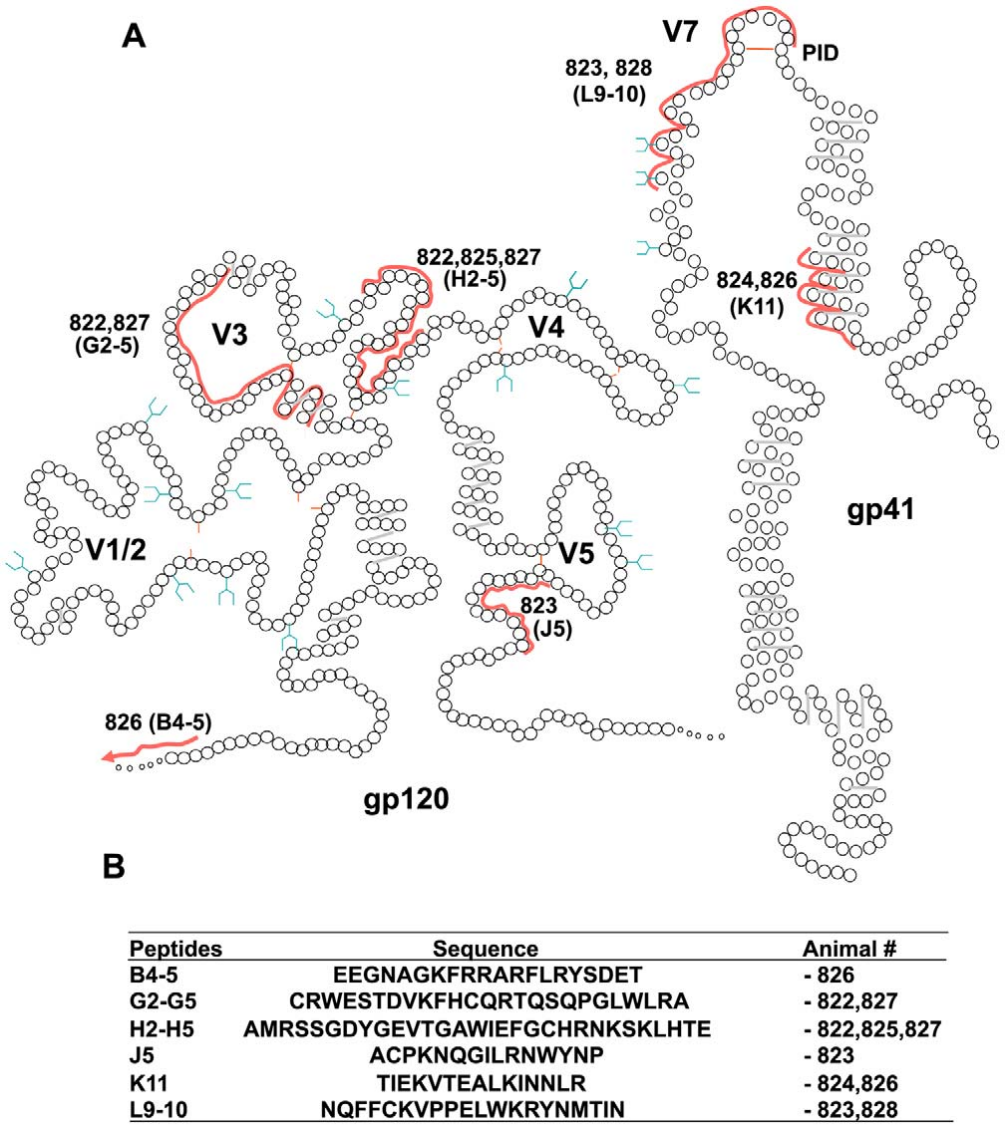
(A) Location of T cell epitopes identified by ELISpot on schematic representations of FIV gp120 and gp41. Each circle represents a single amino acid, regions constituting T cell epitopes are marked by a red line with responding cat number alongside and peptide names in parenthesis. Predicted sites for N-linked glycosylation are in blue. (B) Amino acid sequences of T cell epitopes covered by the ELISpot peptides and the animals that responded.

Resolution of the pools revealed either individual peptides or groups of peptides were responsible for driving the production of IFN-γ; for example, the response of 823 (group 1) to pool P12 could be mapped to peptide J3 while the epitope(s) in pool P9 recognised by 822 (group 1) and 827 (group 2) spanned peptides H2 to H5. Superimposing the amino acid sequences of the peptides recognised upon a schematic representation of FIV gp120 and gp41 [32] (Fig. 8) revealed gp120 epitopes at the leader-surface glycoprotein (L-SU) junction (B4-5), in the V3 loop (G2-5), between V3 and V4 (H2-5) and C-terminal to V5 (J5) while gp41 epitopes mapped to the α-helical region of the extracellular domain (K11) and a large epitope spanning the principle immunodominant domain (PID) [33] and V7 (L9-10) recognised by both 823 (group 1) and 828 (group 2). Three of the Env epitopes were recognised by cells from two animals while a single epitope elicited responses from the cells of three animals, suggesting that these epitopes may be immunodominant. However, we could find no correlation between the location of T cell epitopes and the amino acid substitutions unique to each variant. Additional ELISpot analyses performed using V5 peptides representative of each of the six viral variants revealed no evidence to suggest that the highly variable V5 loop encompassed a T cell epitope (not shown). The data suggest that while robust T cell responses are induced during the early phase of FIV GL8 infection and that they may contribute to the control of viral replication, they are unlikely to account for the selective failure of viral variants such as B14 and B28 to propagate following experimental transmission.

### Sensitivity of the viral variants to receptor antagonists

Of the six variants constituting the quasispecies, B14 achieved the lowest viral load *in vivo*. As a reduced viral load was evident as early as 3 weeks post-infection, it is unlikely that an adaptive immune response was responsible for the poor replication of B14, more likely would be either an increased sensitivity to an innate control mechanism or a reduction in the efficiency of the process of viral entry and spread. Previously, we observed that variants B14 and B28 displayed an increased resistance to inhibition by soluble CD134 coupled with an enhanced sensitivity to inhibition by anti-CD134 antibody ([15]), indicative of alterations in the virus-receptor interaction. For B14, modulation of the virus-CD134 interaction was localised to the acquisition of mutations in the V5 loop, apparently driven by escape from virus neutralising antibodies [15]. Thus the two viral variants that achieved the lowest viral loads *in vivo* also displayed an altered interaction with the primary receptor CD134. We therefore extended these studies to the interaction between the virus and its co-receptor CXCR4. HIV(FIV) pseudotypes bearing Envs from GL8 or the five variants were used to infect either MYA-1 cells (low CXCR4) or CLL-CD134 (high CXCR4) in the presence of increasing concentrations of the CXCR4 antagonist AMD3100 and the 50% inhibitory concentration (IC50) estimated (Table 1). Infection with all six variants was inhibited by AMD3100, consistent with obligate usage of CXCR4 for entry by FIV. Lower concentrations of AMD3100 were required to inhibit infection of cells expressing low levels of CXCR4 (MYA-1) than high levels of CXCR4 (CLL-CD134). Only one viral variant (B14) showed an enhanced sensitivity to antagonism by AMD3100, most markedly on MYA-1 cells where CXCR4 was limiting. Thus, in addition to a high sensitivity to anti-CD134 antibody and a low sensitivity to soluble CD134, B14 was more sensitive to the CXCR4 antagonist. In the absence of evidence for selective humoral or cellular control of the early replication of this viral variant in the infected host, a deficiency in the process of viral entry and spread may account for the reduced ability to infect cells *in vitro* and the poor replication *in vivo*.

**Table 1:** Sensitivity of viral variants to receptor antagonists.

		Viral variant					
	Target cell	B14	B19	B28	B30	B31	B32
anti- CD134^1^	MYA-1	0.11	0.34	0.16	0.26	>50	>50
sFc- CD134	MYA-1	25	1.1	18	1.6	1.4	0.7
	MCC- CD134	40	1.8	20	1.5	1.6	1.3
AMD3100	MYA-1	0.0045	0.06	0.03	0.02	0.035	0.025
	CLL- CD134	0.14	0.4	1.0	0.5	0.5	0.5

Inhibitory concentration 50% (IC50) in µg/ml.

## Discussion

CD134 (OX40) is the primary receptor for FIV [1]; however, viruses differ in the way they interact with CD134 [13], [14] (reviewed in [42]). These differences in the virus-receptor interaction appear to be predictive of sensitivity to antagonism by anti-CD134 antibody and soluble CD134, suggesting that viruses may differ in the specificity and affinity with which they interact with CD134. Previously, we proposed that viruses may evolve *in vivo* from a complex, high affinity CD134 interaction (the predominant phenotype in early, acute infection) to a more simple, low affinity interaction (more prominent in late, chronic infection). Consistent with this hypothesis, we observed the evolution of the receptor usage of the “early” isolate GL8 *in vivo* and the emergence of variants with the characteristics of “late” isolates [15]. If it is indeed the case that GL8-like variants dominate in early infection, then variants with the “late” phenotype must either not be transmitted, or fail to thrive following transmission.

In this study we have addressed the latter scenario, by infecting cats with a reconstituted quasispecies of “early” and “late” variants, we observed the preferential replication of GL8-like “early” variants. The basis for the failure of “late” variants to thrive following transmission did not appear to stem from immunological control; we could find no evidence for humoral control of viral replication during the 21 week study period. Further, while potent Env-specific cellular immune responses were detected and their specificities mapped, there was no correlation between the epitopes targeted and the replication of individual viral variants. However, viruses that failed to thrive following transmission, such as B14 and B28, were more sensitive to antagonism by anti-CD134 antibody, resisted inhibition by soluble CD134, and in the case of B14, displayed an enhanced sensitivity to CXCR4 antagonist. Each of these observations is consistent with alterations in the nature of the virus-receptor interaction in variants such as B14 and B28, for example a reduced affinity of the B14 and B28 Envs for the viral receptors. Our data suggest that these alterations place the “late” variants at a replicative disadvantage in naïve cats.

Previous data have suggested that the neutralising response to the GL8 strain of FIV develops very slowly [28]. In this study, we could find no evidence to suggest that virus neutralising antibodies played a significant role in the control of the GL8 strain of FIV during the first 21 weeks of infection. Potent neutralising responses have been observed in chronically infected cats (reviewed in [28]), however this response may be focussed to a limited number of determinants on Env such as the V4 and V5 loops and escape from neutralisation has been observed [15], [43]–[47]. Using HIV(FIV) pseudotypes bearing the Env proteins from each of the viral variants within the inoculum we established that the strains that replicated weakly *in vivo* were not targeted selectively by neutralising antibodies during the 21 week study period.

Replication of GL8 induced significant cellular immune responses in both study groups by 21 weeks post-infection, the epitopes recognised varying between animals, an observation that would be consistent with the out-bred study group possessing distinct MHC and T cell repertoires. Mapping the cellular responses proved informative: strong responses were detected against epitopes in the V3–V4 region, perhaps indicating that at least some of the pressure for antigenic variation in these regions may come from the cellular immune response. Conversely, no epitopes were detected in V5, the region that is most variable *in vivo*, consistent with previous studies suggesting that this region evolves to escape the humoral response [15], [45]. Although we were able to fine-map the determinants in Env targeted by the early cellular response, no consistent pattern emerged to suggest that cells infected with variants that replicated poorly were targeted more efficiently by the cellular response. Given the potency of the cellular immune responses detected, it is likely that there is a cellular element to controlling FIV GL8 replication during the acute phase of infection but that additional factors act upon variants such as B14 to stem viral spread.

Our data suggest that the variants that emerged late in the infection of cat A613 have a reduced ability to infect diverse cells types and that this may stem from alterations in the way they interact with the viral receptors CXCR4 and CD134. The anti-CD134 antibody 7D6 competes very poorly with Env for binding to CD134, suggesting that the inhibitory effect may be allosteric in nature [48]. GL8-like viruses are relatively resistant to 7D6, perhaps suggesting a higher affinity interaction for CD134 than B14-like variants. Thus GL8-like viruses may simply dominate in early infection as they infect the primary target cells more efficiently. Once an infection has been established, the immunological activation associated with infection exemplified by lymphoid hyperplasia [26], [27], enhanced pro-inflammatory cytokine production [49] and polyclonal B cell activation [50] may result in up-regulation of receptor expression and thus create an environment that is conducive to the emergence of viral variants displaying a reduced requirement for high affinity receptor interactions.

The observation that GL8-like and B14-like viruses differ in the way they interact with the viral receptors CD134 and CXCR4 is consistent with findings that have revealed that HIV-1 clade B envelopes from early infection tend to harbour amino acid signatures that favour efficient Env expression in infected cells, enhancing Env incorporation into nascent virions and replication to higher titres [51], [52]. These signature sequences are lost during chronic infection under selective pressure from the adaptive immune response [51], [52]. *Ex vivo* assays of primary HIV-1 isolates revealed that fitness mapped to the *env* gene and was controlled predominantly during the early stages of viral replication [53], [54]. For example, some type C HIV-1 *envs* displayed reduced fitness in comparison with those of type B isolates and this was associated with weak cell surface binding, inefficient entry, and an increased sensitivity to CCR5 antagonists and fusion inhibitors [55]. By analogy to these studies, we could hypothesize that FIV variants such as B14 and B28 have an altered affinity for the viral receptor or co-receptor that reduces fitness, translating to a lower replicative capacity.

B14-like viruses may represent the end-product of selection by the cellular and humoral immune responses upon FIV GL8; viruses that emerge in chronic infection but which would be supplanted rapidly by GL8-like viruses following transmission. Whether B14-like viruses are able to disseminate or replicate in distinct cellular compartments remains to be established, target populations may include B cells or CD8+ T cells, however reliable systems for the derivation and maintenance of feline CD8+ T cell and B cell lines are not currently available.

The choice of immunogen is an important consideration for the design of novel FIV vaccines. The data gathered from this study would favour immunogens based on strains such as the parent GL8 virus since it is this type of virus that dominates in early infection and is responsible for the majority of the proviral burden during the acute phase. In targeting such viruses selectively, novel vaccines may not induce sterilising immunity, however they would be more likely to prevent the establishment of a high viral burden associated with rapid disease progression. Whether immunogens can be designed that target such variants of FIV and HIV effectively, inducing broad and long-lasting immunity remains one of the greatest challenges in AIDS research.

## Supporting Information

Figure S1 **Location of non-synonymous mutations on the Envs from the variants of GL8.** Variant B32 Env was identical to the GL8414 molecular clone. Yellow circles represent single amino acid changes, solid yellow block represents multiple changes. Each Env is defined by i) sensitivity to neutralisation by postmortem plasma from cat 613; ii) receptor usage (dependency on cysteine rich domains (CRDs) 1 and 2 of CD134; iii) sensitivity to inhibition by anti-CD134 antibody 7D6; and iv) sensitivity to inhibition by soluble CD134.
(PDF)

Figure S2 **Predicted amino acid sequence alignment of the SU-TM region from clones B14, B19, B28, B30, B31, B32 and the parent clone GL8 (414).** The SU-TM encoding region of each env was cloned into the GL8MYA molecular clones using Mlu-I and Nde-I sites at the L-SU junction and RRE respectively. Thus, in all recombinant viruses the L-SU cleavage site is mutated from RRAR to RRVR.
(PDF)

Figure S3 **Study design.** Previously, three animals (A611, A612 and A613) were infected with the GL8(414) molecular clone of FIV and followed for 322 weeks [5]. At post-mortem, a viral quasispecies was identified in the peripheral blood of cat A613. Env genes representative of five viral variants (B14, B19, B28, B30, B31) and the parent virus (B32) were cloned into the GL8 molecular clone and used to prepare i) a homogeneous preparation of GL8 B32 or ii) a reconstituted quasispecies comprising equal amounts of B14, B19, B28, B30, B31 and B32. Two groups of four animals were infected with matched TCID50 of the two stocks and monitored for 21 weeks, at which time the study was terminated and postmortem analyses performed. A821 died mid-study as a result of a condition unrelated to FIV infection.
(PDF)

## References

[1] Shimojima M, Miyazawa T, Ikeda Y, McMonagle EL, Haining H, et al (2004) Use of CD134 as a primary receptor by the feline immunodeficiency virus. Science 303: 1192–1195. 14976315 10.1126/science.1092124

[2] de Parseval A, Chatterji U, Sun P, Elder JH (2004) Feline immunodeficiency virus targets activated CD4+ T cells by using CD134 as a binding receptor. ProcNatlAcadSciUSA 101: 13044–13049. 10.1073/pnas.0404006101 PMC516514 15326292

[3] Willett BJ, Hosie MJ, Neil JC, Turner JD, Hoxie JA (1997) Common mechanism of infection by lentiviruses. Nature 385: 587. 9024654 10.1038/385587a0

[4] Willett BJ, Picard L, Hosie MJ, Turner JD, Adema K, et al (1997) Shared usage of the chemokine receptor CXCR4 by the feline and human immunodeficiency viruses. Journal of Virology 71: 6407–6415. 9261358 10.1128/jvi.71.9.6407-6415.1997 PMC191914

[5] Hosie MJ, Willett BJ, Klein D, Dunsford TH, Cannon C, et al (2002) Evolution of replication efficiency following infection with a molecularly cloned feline immunodeficiency virus of low virulence. Journal of Virology 76: 6062–6072. 12021339 10.1128/JVI.76.12.6062-6072.2002 PMC136200

[6] Diehl LJ, Mathiason-Dubard CK, O'Neil LL, Obert LA, Hoover EA (1995) Induction of accelerated feline immunodeficiency virus disease by acute-phase virus passage. Journal of Virology 69: 6149–6157. 7666517 10.1128/jvi.69.10.6149-6157.1995 PMC189512

[7] Inoshima Y, Kohmoto M, Ikeda Y, Yamada H, Kawaguchi Y, et al (1996) Roles of the auxiliary genes and AP-1 binding site in the long terminal repeat of feline immunodeficiency virus in the early stage of infection in cats. Journal of Virology 70: 8518–8526. 8970975 10.1128/jvi.70.12.8518-8526.1996 PMC190943

[8] Dean GA, Himathongkham S, Sparger EE (1999) Differential cell tropism of feline immunodeficiency virus molecular clones in vivo. Journal of Virology 73: 2596–2603. 10074104 10.1128/jvi.73.4.2596-2603.1999 PMC104014

[9] Pistello M, Moscardini M, Mazzetti P, Bonci F, Zaccaro L, et al (2002) Development of feline immunodeficiency virus ORF-A (tat) mutants: in vitro and in vivo characterization. Virology 298: 84–95. 12093176 10.1006/viro.2002.1442

[10] Munk C, Beck T, Zielonka J, Hotz-Wagenblatt A, Chareza S, et al (2008) Functions, structure, and read-through alternative splicing of feline APOBEC3 genes. Genome Biol 9: R48. 18315870 10.1186/gb-2008-9-3-r48 PMC2397500

[11] Dean GA, Reubel GH, Moore PF, Pedersen NC (1996) Proviral burden and infection kinetics of feline immunodeficiency virus in lymphocyte subsets of blood and lymph node. Journal of Virology 70: 5165–5169. 8764024 10.1128/jvi.70.8.5165-5169.1996 PMC190471

[12] English RV, Johnson CM, Gebhard DH, Tompkins MB (1993) In vivo lymphocyte tropism of feline immunodeficiency virus. Journal of Virology 67: 5175–5186. 7688819 10.1128/jvi.67.9.5175-5186.1993 PMC237915

[13] Willett BJ, McMonagle EL, Ridha S, Hosie MJ (2006) Differential utilization of CD134 as a functional receptor by diverse strains of feline immunodeficiency virus (FIV). Journal of Virology 80: 3386–3394. 16537606 10.1128/JVI.80.7.3386-3394.2006 PMC1440405

[14] Willett BJ, McMonagle EL, Bonci F, Pistello M, Hosie MJ (2006) Mapping the domains of CD134 as a functional receptor for feline immunodeficiency virus. Journal of Virology 80: 7744–7747. 16840353 10.1128/JVI.00722-06 PMC1563730

[15] Willett BJ, Kraase M, logan N, McMonagle EL, Samman A, et al (2010) Modulation of the virus-receptor interaction by mutations in the V5 loop of feline immunodeficiency virus (FIV) following in vivo escape from neutralising antibody. Retrovirology 7: 38. 20420700 10.1186/1742-4690-7-38 PMC2873508

[16] Connor RI, Sheridan KE, Ceradini D, Choe S, Landau NR (1997) Change in coreceptor use correlates with disease progression in HIV- 1-infected individuals. Journal of Experimental Medicine 185: 621–628. 9034141 10.1084/jem.185.4.621 PMC2196142

[17] Margolis L, Shattock R (2006) Selective transmission of CCR5-utilizing HIV-1: the ‘gatekeeper’ problem resolved? NatRevMicrobiol 4: 312–317. 10.1038/nrmicro1387 16541138

[18] Hosie M, Osborne R, Yamamoto JK, Neil JC, Jarrett O (1995) Protection against homologous but not heterologous challenge induced by inactivated feline immunodeficiency virus vaccines. Journal of Virology 69: 1253–1255. 7815500 10.1128/jvi.69.2.1253-1255.1995 PMC188698

[19] Pu R, Coleman J, Coisman J, Sato E, Tanabe T, et al (2005) Dual-subtype FIV vaccine (Fel-O-Vax FIV) protection against a heterologous subtype B FIV isolate. JFelineMedSurg 7: 65–70. 10.1016/j.jfms.2004.08.005 PMC10911555 15686976

[20] Pu R, Coleman J, Omori M, Arai M, Hohdatsu T, et al (2001) Dual-subtype FIV vaccine protects cats against in vivo swarms of both homologous and heterologous subtype FIV isolates. AIDS 15: 1225–1237. 11426067 10.1097/00002030-200107060-00004

[21] Yamamoto JK, Hohdatsu T, Olmsted RA, Pu R, Louie H, et al (1993) Experimental vaccine protection against homologous and heterologous strains of feline immunodeficiency virus. Journal of Virology 67: 601–605. 8380095 10.1128/jvi.67.1.601-605.1993 PMC237403

[22] Yamamoto JK, Okuda T, Ackley CD, Louie H, Pembroke E, et al (1991) Experimental vaccine protection against feline immunodeficiency virus. Aids Research and Human Retroviruses 7: 911–921. 1662057 10.1089/aid.1991.7.911

[23] Dunham SP, Bruce J, MacKay S, Golder M, Jarrett O, et al (2006) Limited efficacy of an inactivated feline immunodeficiency virus vaccine. Veterinary Record 158: 561–562. 16632531 10.1136/vr.158.16.561

[24] Callanan JJ, Jones BA, Irvine J, Willett BJ, McCandlish IAP, et al (1996) Histological classification and immunophenotype of lymphosarcomas in cats with naturally and experimentally acquired feline immunodeficiency virus infections. Veterinary Pathology 33: 264–272. 8740699 10.1177/030098589603300302

[25] Callanan JJ, McCandlish IAP, O'Neil B, Lawrence CE, Rigby M, et al (1992) Lymphosarcoma in experimentally induced feline immunodeficiency virus infection. Veterinary Record 130: 293–295. 1317615 10.1136/vr.130.14.293

[26] Callanan JJ, Racz P, Thompson H, Jarrett O (1993) Morphologic characterization of the lymph node changes in feline immunodeficiency virus infection as an animal model of AIDS. In: Racz P, Letvin NL, Gluckman JC, editors. Animal models of HIV and other retroviral infections. Basel, Switzerland: S. Karger. 115–136.

[27] Callanan JJ, Thompson H, Toth SR, O'Neil B, Lawrence CE, et al (1992) Clinical and pathological findings in feline immunodeficiency virus experimental infection. Veterinary Immunology and Immunopathology 35: 3–13. 1337400 10.1016/0165-2427(92)90116-8 PMC7119604

[28] Hosie MJ, Pajek D, Samman A, Willett BJ (2011) Feline Immunodeficiency Virus (FIV) Neutralization: A Review. Viruses 3: 1870–1890. 22069520 10.3390/v3101870 PMC3205386

[29] Kraase M, Sloan R, Klein D, logan N, McMonagle L, et al (2010) Feline immunodeficiency virus env gene evolution in experimentally infected cats. Veterinary Immunology and Immunopathology 134: 96–106. 19897254 10.1016/j.vetimm.2009.10.015

[30] Miyazawa TM, Furuya T, Itagaki S, Tohya Y, Takahashi E, et al (1989) Establishment of a feline T-lymphoblastoid cell line highly sensitive for replication of feline immunodeficiency virus. Archives of Virology 108: 131–135. 2480760 10.1007/BF01313750

[31] Willett BJ, Hosie MJ, Callanan JJ, Neil JC, Jarrett O (1993) Infection with feline immunodeficiency virus is followed by the rapid expansion of a CD8+ lymphocyte subset. Immunology 78: 1–6. 8094707 PMC1421779

[32] Laemmli UK (1970) Cleavage of structural proteins during the assembly of the head of bacteriophage T4. Nature 227: 680–685. 5432063 10.1038/227680a0

[33] Ryan G, Klein D, Knapp E, Hosie MJ, Grimes T, et al (2003) Dynamics of viral and proviral loads of feline immunodeficiency virus within the feline central nervous system during the acute phase following intravenous infection. Journal of Virology 77: 7477–7485. 12805447 10.1128/JVI.77.13.7477-7485.2003 PMC164807

[34] Koup RA, Safrit JT, Cao Y, Andrews CA, McLeod G, et al (1994) Temporal association of cellular immune responses with the initial control of viremia in primary human immunodeficiency virus type 1 syndrome. Journal of Virology 68: 4650–4655. 8207839 10.1128/jvi.68.7.4650-4655.1994 PMC236393

[35] Borrow P, Lewicki H, Hahn BH, Shaw GM, Oldstone MB (1994) Virus-specific CD8+ cytotoxic T-lymphocyte activity associated with control of viremia in primary human immunodeficiency virus type 1 infection. Journal of Virology 68: 6103–6110. 8057491 10.1128/jvi.68.9.6103-6110.1994 PMC237022

[36] Wilson JD, Ogg GS, Allen RL, Davis C, Shaunak S, et al (2000) Direct visualization of HIV-1-specific cytotoxic T lymphocytes during primary infection. AIDS 14: 225–233. 10716497 10.1097/00002030-200002180-00003

[37] Pantaleo G, Demarest JF, Soudeyns H, Graziosi C, Denis F, et al (1994) Major expansion of CD8+ T cells with a predominant V beta usage during the primary immune response to HIV. Nature 370: 463–467. 8047166 10.1038/370463a0

[38] Nishimura Y, Shimojima M, Sato E, Izumiya Y, Tohya Y, et al (2004) Downmodulation of CD3epsilon expression in CD8alpha+beta- T cells of feline immunodeficiency virus-infected cats. Journal of General Virology 85: 2585–2589. 15302952 10.1099/vir.0.80102-0

[39] Shimojima M, Nishimura Y, Miyazawa T, Tohya Y, Akashi H (2003) Phenotypic changes in CD8+ peripheral blood lymphocytes in cats infected with feline immunodeficiency virus. MicrobesInfect 5: 1171–1176. 10.1016/j.micinf.2003.08.004 14623012

[40] Paillot R, Richard S, Bloas F, Piras F, Poulet H, et al (2005) Toward a detailed characterization of feline immunodeficiency virus-specific T cell immune responses and mediated immune disorders. Veterinary Immunology and Immunopathology 106: 1–14. 15910988 10.1016/j.vetimm.2004.12.023

[41] Dean GA, Lavoy A, Burkhard MJ (2004) Peptide mapping of feline immunodeficiency virus by IFN-gamma ELISPOT. Veterinary Immunology and Immunopathology 100: 49–59. 15182995 10.1016/j.vetimm.2004.03.001

[42] Willett BJ, Hosie MJ (2008) Chemokine receptors and co-stimulatory molecules: Unravelling feline immunodeficiency virus infection. Veterinary Immunology and Immunopathology 123: 56–64. 18289703 10.1016/j.vetimm.2008.01.012 PMC2413005

[43] Bendinelli M, Pistello M, Del Mauro D, Cammarota G, Maggi F, et al (2001) During readaptation in vivo, a tissue culture-adapted strain of feline immunodeficiency virus reverts to broad neutralization resistance at different times in individual hosts but through changes at the same position of the surface glycoprotein. Journal of Virology 75: 4584–4593. 11312328 10.1128/JVI.75.10.4584-4593.2001 PMC114211

[44] Giannecchini S, Matteucci D, Ferrari A, Pistello M, Bendinelli M (2001) Feline immunodeficiency virus-infected cat sera associated with the development of broad neutralization resistance in vivo drive similar reversions in vitro. Journal of Virology 75: 8868–8873. 11507234 10.1128/JVI.75.18.8868-8873.2001 PMC115134

[45] Samman A, Iogan N, McMonagle EL, Ishida T, Mochizuki M, et al (2010) Neutralization of feline immunodeficiency virus by antibodies targeting the V5 loop of Env. Journal of General Virology 91: 242–249. 19776242 10.1099/vir.0.015404-0

[46] Siebelink KH, Bosch ML, Rimmelzwaan GF, Meloen RH, Osterhaus AD (1995) Two different mutations in the envelope protein of feline immunodeficiency virus allow the virus to escape from neutralization by feline serum antibodies. Veterinary Immunology and Immunopathology 46: 51–59. 7542412 10.1016/0165-2427(94)07005-r

[47] Siebelink KHJ, Huisman W, Karlas JA, Rimmelzwaan GF, Bosch ML, et al (1996) Neutralization of feline immunodeficiency virus by polyclonal feline antibody: simultaneous involvement of hypervariable regions 4 and 5 of the surface glycoprotein. Journal of Virology 69: 5124–5127. 10.1128/jvi.69.8.5124-5127.1995 PMC189331 7609081

[48] Willett BJ, McMonagle EL, logan N, Spiller OB, Schneider P, et al (2007) Probing the interaction between the feline immunodeficiency virus and CD134 using a novel monoclonal antibody 7D6 and CD134L (OX40L). Journal of Virology 81: 9665–9679. 17609274 10.1128/JVI.01020-07 PMC2045395

[49] Lawrence CE, Callanan JJ, Willett BJ, Jarrett O (1995) Cytokine production by cats infected with FIV: a longitudinal study. Immunology 85: 568–574. 7558151 PMC1383785

[50] Flynn JN, Cannon CA, Lawrence CE, Jarrett O (1994) Polyclonal B-cell activation in cats infected with feline immunodeficiency virus. Immunology 81: 626–630. 7518798 PMC1422366

[51] Asmal M, Hellmann I, Liu WM, Keele BF, Perelson AS, et al.. (2011) A Signature in HIV-1 Envelope Leader Peptide Associated with Transition from Acute to Chronic Infection Impacts Envelope Processing and Infectivity. PLoS One 6. 10.1371/journal.pone.0023673 PMC3158090 21876761

[52] Gnanakaran S, Bhattacharya T, Daniels M, Keele BF, Hraber PT, et al.. (2011) Recurrent Signature Patterns in HIV-1 B Clade Envelope Glycoproteins Associated with either Early or Chronic Infections. Plos Pathogens 7. 10.1371/journal.ppat.1002209 PMC3182927 21980282

[53] Rangel HR, Weber J, Chakraborty B, Gutierrez A, Marotta ML, et al (2003) Role of the human immunodeficiency virus type 1 envelope gene in viral fitness. J Virol 77: 9069–9073. 12885922 10.1128/JVI.77.16.9069-9073.2003 PMC167250

[54] Ball SC, Abraha A, Collins KR, Marozsan AJ, Baird H, et al (2003) Comparing the ex vivo fitness of CCR5-tropic human immunodeficiency virus type 1 isolates of subtypes B and C. J Virol. 77: 1021–1038. 10.1128/JVI.77.2.1021-1038.2003 PMC140829 12502818

[55] Marozsan AJ, Moore DM, Lobritz MA, Fraundorf E, Abraha A, et al (2005) Differences in the fitness of two diverse wild-type human immunodeficiency virus type 1 isolates are related to the efficiency of cell binding and entry. J Virol 79: 7121–7134. 15890952 10.1128/JVI.79.11.7121-7134.2005 PMC1112120

