# Supplementary material for: Selective Expansion of Viral Variants following Experimental Transmission of a Reconstituted Feline Immunodeficiency Virus Quasispecies
Source: PLoS One. 2013 Jan 23;8(1):e54871. doi: 10.1371/journal.pone.0054871 (PMC3553009; doi:10.1371/journal.pone.0054871)
Supplement: Figure S1 — Location of non-synonymous mutations on the Envs from the variants of GL8. Variant B32 Env was identical to the GL8414 molecular clone. Yellow circles represent single amino acid changes, solid yellow block represents multiple changes. Each Env is defined by i) sensitivity to neutralisation by postmortem plasma from cat 613; ii) receptor usage (dependency on cysteine rich domains (CRDs) 1 and 2 of CD134; iii) sensitivity to inhibition by anti-CD134 antibody 7D6; and iv) sensitivity to inhibition by soluble CD134. (PDF) [file pone.0054871.s001.pdf]

B14

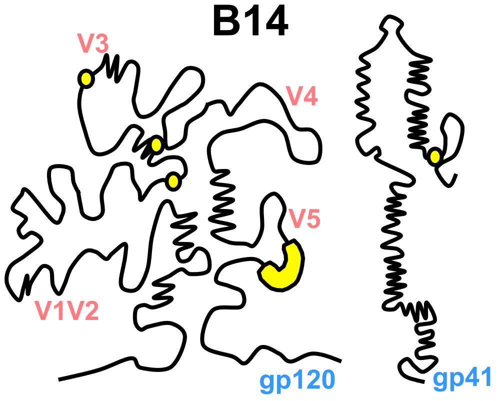

V5 NAb resistant
CRD1
Anti-CD134 sensitive
Soluble CD134 resistant

B19

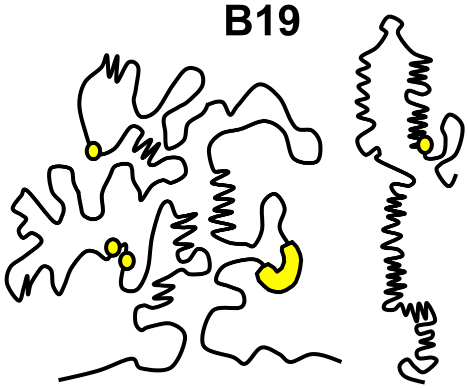

V5 NAb resistant
CRD1
Anti-CD134 intermediate
Soluble CD134 sensitive

B28

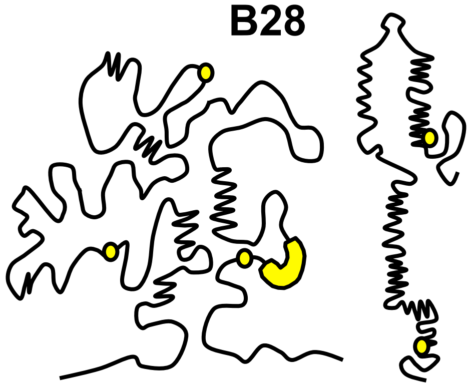

V5 NAb resistant
CRD1
Anti-CD134 sensitive
Soluble CD134 resistant

B30

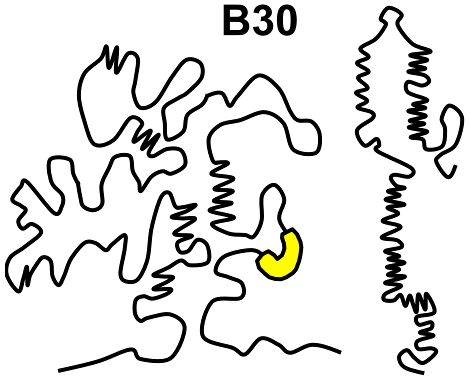

V5 NAb resistant
CRD1
Anti-CD134 intermediate
Soluble CD134 sensitive

B31

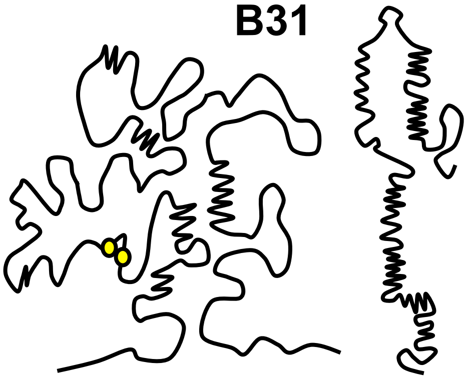

V5 NAb sensitive
CRD1+CRD2
Anti-CD134 resistant
Soluble CD134 sensitive

B32

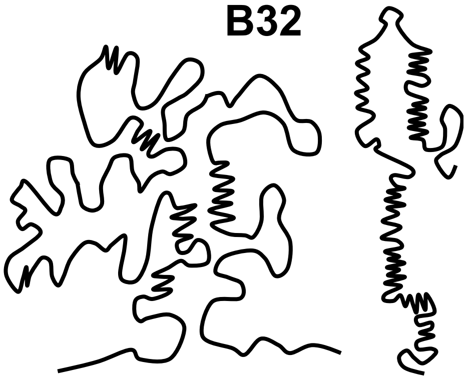

V5 NAb sensitive
CRD1+CRD2
Anti-CD134 resistant
Soluble CD134 sensitive
