# Supplementary material for: Selective Expansion of Viral Variants following Experimental Transmission of a Reconstituted Feline Immunodeficiency Virus Quasispecies
Source: PLoS One. 2013 Jan 23;8(1):e54871. doi: 10.1371/journal.pone.0054871 (PMC3553009; doi:10.1371/journal.pone.0054871)
Supplement: Figure S2 — Predicted amino acid sequence alignment of the SU-TM region from clones B14, B19, B28, B30, B31, B32 and the parent clone GL8 (414). The SU-TM encoding region of each env was cloned into the GL8MYA molecular clones using Mlu-I and Nde-I sites at the L-SU junction and RRE respectively. Thus, in all recombinant viruses the L-SU cleavage site is mutated from RRAR to RRVR. (PDF) [file pone.0054871.s002.pdf]

```
GL8414    RRARFLRYSDETILSLIHLFIGYCTYLLNRKELGSLRHDIDIEAPQEECYSSREQGITDNIKYGKRCF  68
B14       RRVRFLRYSDETILSLIHLFIGYCTYLLNRKELGSLRHDIDIEAPQEECYSSREQGITDNIKYGKRCF  68
B19       RRVRFLRYSDETILSLIHLFIGYCTYLLNRKELGSLRHDIDIEAPQEECYSSREQGITDNIKYGKRCF  68
B28       RRVRFLRYSDETILSLIHLFIGYCTYLLNRKELGSLRHDIDIEAPQEECYSSREQGITDNIKYGKRCF  68
B31       RRVRFLRYSDETILSLIHLFIGYCTYLLNRKELGSLRHDIDIEAPQEECYSSREQGITDNIKYGKRCF  68
B30       RRVRFLRYSDETILSLIHLFIGYCTYLLNRKELGSLRHDIDIEAPQEECYSSREQGITDNIKYGKRCF  68
B32       RRVRFLRYSDETILSLIHLFIGYCTYLLNRKELGSLRHDIDIEAPQEECYSSREQGITDNIKYGKRCF  68
          **.*****************************************************************

GL8414    IGTAGLYLLLFIGVGIYLGTAKAQVVWRLPPLVVPVEESEIIFWDCWAPEEPACQDFLGAMIHLKAST  136
B14       IGTAGLYLLLFIVG-IYWGTAKAQVVWRLPPLVVPVEESEIIFWDCWAPEEPACQDFLGAMIHLKAST  135
B19       IGTAGLYLLLFIGVGIYLGTAKAQVVWRLPPLVVPVEESEIIFWDCWAPEEPACQDFLGAMIHLKAST  136
B28       IGTAGLYLLLFIG--IYLGTAKAQVVWRLPPLVVPVEESEIIFWDCWAPEEPACQDFLGAMIHLKAST  134
B31       IGTAGLYLLLFIGVGIYLGTAKAQVVWRLPPLVVPVEESEIIFWDCWAPEEPACQDFLGAMIHLKAST  136
B30       IGTAGLYLLLFIG--IYLGTAKAQVVWRLPPLVVPVEESEIIFWDCWAPEEPACQDFLGAMIHLKAST  134
B32       IGTAGLYLLLFIGVGIYLGTAKAQVVWRLPPLVVPVEESEIIFWDCWAPEEPACQDFLGAMIHLKAST  136
          ************   ** **************************************************

GL8414    NISIQEGPTLGNWAKEIWGTLFKKATRQCRRGRIWKRWNETITGPLGCANNTCYNISVIVPDYQCYLD  204
B14       NISIQEGPTLGNWAKEIWGTLFKKATRQCRRGRIWKRWNETITGPLGCANNTCYNISVIVPDYQCYLD  203
B19       NISIQEGPTLGNWAKEIWGTLFKKATRQCRRGRIWKRWNETITGPSGCAKNTCYNISVIVPDYQCYLD  204
B28       NISIQEGPTLGNWAKEIWGTLFKKATRQCRRGRIWKRWNETITGPLGCDNNTCYNISVIVPDYQCYLD  202
B31       NISIQEGPTLGNWAKEIWGTLFKKATRQCRRGRVWKRWNETITGPSGCAANTCYNISVIVPDYQCYLD  204
B30       NISIQEGPTLGNWAKEIWGTLFKKATRQCRRGRIWKRWNETITGPLGCANNTCYNISVIVPDYQCYLD  202
B32       NISIQEGPTLGNWAKEIWGTLFKKATRQCRRGRIWKRWNETITGPLGCANNTCYNISVIVPDYQCYLD  204
          *********************************:*********** **  ******************

GL8414    RVDTWLQGKVNVSLCLTGGKMLYNKYTKQLSYCTDPLQIPLINYTFGPNQTCMWNTSQIQDPEIPKCG  272
B14       RVDTWLQGKVNVSLCLTGGKMLYNKYTKQLSYCTDPLQIPLINYTFGPNQTCMWNTSQIQDPEIPKCG  271
B19       RVDTWLQGKVNVSLCLTGGKMLYNKYTKQLSYCTDPLQIPLINYTFGPNQTCMWNTSQIQDPEIPKCG  272
B28       RVDTWLQGKVNVSLCLTGGKMLYNKYTKQLSYCTDPLQIPLINYTFGPNQTCMWNTSQIQDPEIPKCG  270
B31       RVDTWLQGKVNVSLCLTGGKMLYNKYTKQLSYCTDPLQIPLINYTFGPNQTCMWNTSQIQDPEIPKCG  272
B30       RVDTWLQGKVNVSLCLTGGKMLYNKYTKQLSYCTDPLQIPLINYTFGPNQTCMWNTSQIQDPEIPKCG  270
B32       RVDTWLQGKVNVSLCLTGGKMLYNKYTKQLSYCTDPLQIPLINYTFGPNQTCMWNTSQIQDPEIPKCG  272
          ********************************************************************

GL8414    WWNQIAYYNSCRWESTDVKFHCQRTQSQPGLWLRAISSWKQRNRWEWRPDFESEKVKVSLQCNSTKNL  340
B14       WWNQMAYYNSCRWENTDVKFHCQRTQSQPGLWLRAISSWKQGNRWEWRPDFESEKVKVSLQCNSTKNL  339
B19       WWNQIAYYNSCRWESTDVKFHCQRTQSQPGSWLRAISSWKQRNRWEWRPDYESEKVKVSLQCNSTKNL  340
B28       WWNQIAYYNSCRWESTDVKFHCQRTQSQPGLWLRAISSWKQRNRWEWRPDFESEKVKVSLQCNSTKNL  338
B31       WWNQIAYYNSCRWESTDVKFHCQRTQSQPGLWLRAISSWKQRNRWEWRPDFESEKVKVSLQCNSTKNL  340
B30       WWNQIAYYNSCRWESTDVKFHCQRTQSQPGLWLRAISSWKQRNRWEWRPDFESEKVKVSLQCNSTKNL  338
B32       WWNQIAYYNSCRWESTDVKFHCQRTQSQPGLWLRAISSWKQRNRWEWRPDFESEKVKVSLQCNSTKNL  340
          ****:*********.*************** ********** ********:*****************

GL8414    TFAMRSSGDYGEVTGAWIEFGCHRNKSKLHTEARFRIRCRWNVGDNTSLIDTCGETQNVSGANPVDCT  408
B14       TFAMRSSGDYGEVTGAWIEFGCHRNKSKLHTEARFRIRCRWNVGDNTSLIDTCGETQNVSGANPVDCT  407
B19       TFAMRSSGDYGEVTGAWIEFGCHRNKSKLHTEARFRIRCRWNVGDNTSLIDTCGETQNVSGANPVDCT  408
B28       TFAMRSSEDYGEVTGAWIEFGCHRNKSKLHTEARFRIRCRWNVGDNTSLIDTCGETQNVSGANPVDCT  406
B31       TFAMRSSGDYGEVTGAWIEFGCHRNKSKLHTEARFRIRCRWNVGDNTSLIDTCGETQNVSGANPVDCT  408
B30       TFAMRSSGDYGEVTGAWIEFGCHRNKSKLHTEARFRIRCRWNVGDNTSLIDTCGETQKVSGANPVDCT  406
B32       TFAMRSSGDYGEVTGAWIEFGCHRNKSKLHTEARFRIRCRWNVGDNTSLIDTCGETQNVSGANPVDCT  408
          ******* *************************************************:**********

GL8414    MYANRMYNCSLQNGFTMKVDDLIMHFNMTKAVEMYNIAGNWSCTSDLPPTWGYMNCNCTNSSSTN---  473
B14       MYANRMYNCSLQNGFTMKVDDLIMHFNMTKAVEMYNIAGNWSCTSDLPPTWGYMNCNCTNSSSTN---  472
B19       MYANRMYNCSLQNGFTMKVDDLIMHFNMTKAVEMYNIAGNWSCTSDLPPTWGYMNCNCTN-SSTN---  472
B28       MYANRMYNCSLQNGFTMKVDDLIMHFNMTKAVEMYNIAGNWSCTSDLPPTWGYMNCNCTNSSSTNRQN  474
B31       MYANRMYNCSLQNGFTMKVDDLIMHFNMTKAVEMYNIAGNWSCTSDLPPTWGYMNCNCTNSSSTN---  473
B30       MYANRMYNCSLQNGFTMKVDDLIMHFNMTKAVEMYNIAGNWSCTSDLPPTWGYMNCNCTNSSSTN---  471
B32       MYANRMYNCSLQNGFTMKVDDLIMHFNMTKAVEMYNIAGNWSCTSDLPPTWGYMNCNCTNSSSTN---  473
          ************************************************************ ****

GL8414    -----SVKMACPKNQGILRNWYNPVAGLRQSLEKYQVVKQPDYLVVPGEVMEYKPRRKRAAIHVMLAL  536
B14       SSSTNSGEMACPKNQGILRNWYNPVAGLRQSLEKYQVVKQPDYLVVPGEVMEYKPRRKRAAIHVMLAL  540
B19       -----SGKMACPKNQGILRNWYNPVAGLRQSLEKYQVVKQPDYLVVPGEVMEYKPRRKRAAIHVMLAL  535
B28       SSSTNSVKMACPKNQGILRNWYNPVAGLRQSLEKYQVVKQPDYLVVPGEVMEYKPRRKRAAIHVMLAL  542
B31       -----SVKMACPKNQGILRNWYNPVAGLRQSLEKYQVVKQPDYLVVPGEVMEYKPRRKRAAIHVMLAL  536
B30       --STNSVKMACPKNQGILRNWYNPVAGLRQSLEKYQVVKQPDYLVVPGEVMEYKPRRKRAAIHVMLAL  537
B32       -----SVKMACPKNQGILRNWYNPVAGLRQSLEKYQVVKQPDYLVVPGEVMEYKPRRKRAAIHVMLAL  536
               * :************************************************************
```

```
GL8414    ATVLSMAGAGTGATAIGMVTQYHQVLATHQETIEKVTEALKINNLRLVTLEHQVLVIGLKVEAMEKFL  604
B14       ATVLSMAGAGTGATAIGMVTQYHQVLATNQETIEKVTEALKINNLRLVTLEHQVLVIGLKVEAMEKFL  608
B19       ATVLSMAGAGTGATAIGMVTQYHQVLATHQETIEKMTEALKINNLRLVTLEHQVLVIGLKVEAMEKFL  603
B28       ATVLSMAGAGTGATAIGMVTQYHQVLATHQETIEKMTEALKINNLRLVTLEHQVLVIGLKVEAMEKFL  610
B31       ATVLSMAGAGTGATAIGMVTQYHQVLATHQETIEKVTEALKINNLRLVTLEHQVLVIGLKVEAMEKFL  604
B30       ATVLSMAGAGTGATAIGMVTQYHQVLATHQETIEKMTEALKINNLRLVTLEHQVLVIGLKVEAMEKFL  605
B32       ATVLSMAGAGTGATAIGMVTQYHQVLATHQETIEKVTEALKINNLRLVTLEHQVLVIGLKVEAMEKFL  604
          *****************************:*****:********************************

GL8414    YTAFAMQELGCNQNQFFCKVPPELWKRYNMTINQTIWNHGNITLGEWYNQTKELQQKFYEIIMNIEQN  672
B14       YTAFAMQELGCNQNQFFCKVPPELWRRYNMTINQTIWNHGNITLGEWYNQTKELQQKFYEIIMNIEQN  676
B19       YTAFAMQELGCNQNQFFCKVPPELWKRYNMTINQTIWNHGNITLGEWYNQTKELQQKFYEIIMNIEQN  671
B28       YTAFAMQELGCNQNQFFCKVPPELWKRYNMTINQTIWNHGNITLGEWYNQTKELQQKFYEIIMNIEQN  678
B31       YTAFAMQELGCNQNQFFCKVPPELWKRYNMTINQTIWNHGNITLGEWYNQTKELQQKFYEIIMNIEQN  672
B30       YTAFAMQELGCNQNQFFCKVPPELWKRYNMTINQTIWNHGNITLGEWYNQTKELQQKFYEIIMNIEQN  673
B32       YTAFAMQELGCNQNQFFCKVPPELWKRYNMTINQTIWNHGNITLGEWYNQTKELQQKFYEIIMNIEQN  672
          *************************:******************************************

GL8414    NVQGKKGLQQLQEWEDWVGWIGNIPQYLKGLLGGILGIGLGILLLILCLPTLVDCIRNCISKVLGYTV  740
B14       NVQGKKGLQQLQEWEDWVGWIGNIPQYLKGLLGGILGIGLGILLLILCLPTLVDCIRNCISKVLGYTV  744
B19       NVQGKKGLQQLQEWEDWVGWIGNIPQYLKGLLGGILGIGLGILLLILCLPTLVDCIRNCISKVLGYTV  739
B28       NVQGKKGLQQLQEWEDWVGWIGNIPQYLKGLLGGILGIGLGILLLILCLPTLVDCIRNCISKVLGYTV  746
B31       NVQGKKGLQQLQEWEDWVGWIGNIPQYLKGLLGGILGIGLGILLLILCLPTLVDCIRNCISKVLGYTV  740
B30       NVQGKKGLQQLQEWEDWVGWIGNIPQYLKGLLGGILGIGLGILLLILCLPTLVDCIRNCISKVLGYTV  741
B32       NVQGKKGLQQLQEWEDWVGWIGNIPQYLKGLLGGILGIGLGILLLILCLPTLVDCIRNCISKVLGYTV  740
          ********************************************************************

GL8414    IAMPEIDDEEET-VQMELRKNGRQCGMSEKEEE  772
B14       IAMPEIDDEEET-VQMELRKNGRQCGMSEKEEE  776
B19       IAMPEIDDEEE--VQMELRKNGRQCGMSEKEEE  770
B28       IAMPEIDDEEE--VQMELRKNGRQCGMSEKEEE  777
B31       IAMPEIDDEEE--VQMELRKNGRQCGMSEKEEE  771
B30       IAMPEIDDEEEKTVQMELRKNGRQCGMSEKEEE  774
B32       IAMPEIDDEEE--VQMELRKNGRQCGMSEKEEE  771
          ***********  ********************
```
