# Supplementary figures and images for: Selective Expansion of Viral Variants following Experimental Transmission of a Reconstituted Feline Immunodeficiency Virus Quasispecies
Source: PLoS One. 2013 Jan 23;8(1):e54871. doi: 10.1371/journal.pone.0054871 (PMC3553009; doi:10.1371/journal.pone.0054871)

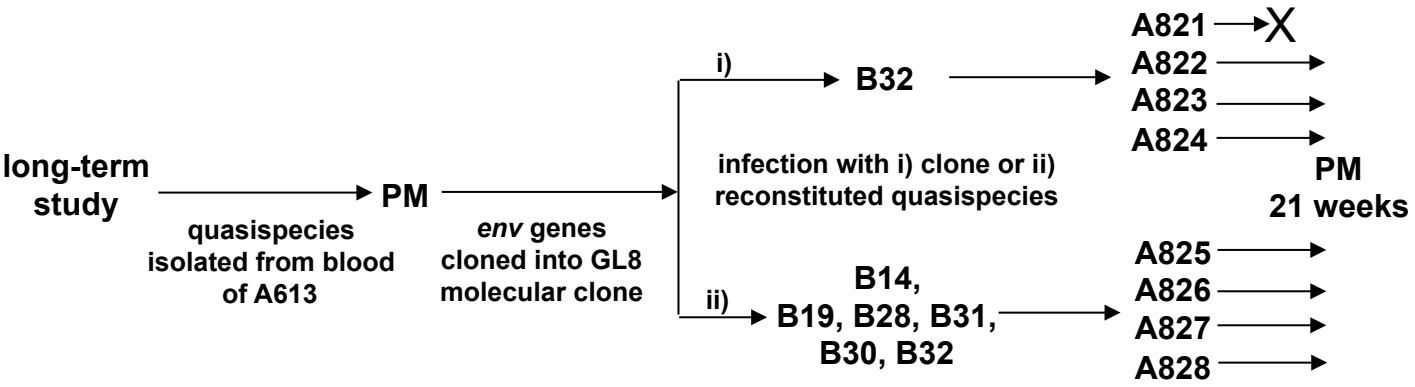

Supplement: Figure S3 — Study design. Previously, three animals (A611, A612 and A613) were infected with the GL8(414) molecular clone of FIV and followed for 322 weeks [5]. At post-mortem, a viral quasispecies was identified in the peripheral blood of cat A613. Env genes representative of five viral variants (B14, B19, B28, B30, B31) and the parent virus (B32) were cloned into the GL8 molecular clone and used to prepare i) a homogeneous preparation of GL8 B32 or ii) a reconstituted quasispecies comprising equal amounts of B14, B19, B28, B30, B31 and B32. Two groups of four animals were infected with matched TCID50 of the two stocks and monitored for 21 weeks, at which time the study was terminated and postmortem analyses performed. A821 died mid-study as a result of a condition unrelated to FIV infection. (PDF) [file pone.0054871.s003.pdf]
